# Global population genomics of the forest pathogen *Dothistroma septosporum *reveal chromosome duplications in high dothistromin‐producing strains

**DOI:** 10.1111/mpp.12791

**Published:** 2019-04-01

**Authors:** Rosie E. Bradshaw, Andre D. Sim, Pranav Chettri, Pierre‐Yves Dupont, Yanan Guo, Lukas Hunziker, Rebecca L. McDougal, Ariska Van der Nest, Arista Fourie, David Wheeler, Murray P. Cox, Irene Barnes

**Affiliations:** ^1^ School of Fundamental Sciences and Bio‐Protection Research Centre Massey University Palmerston North 4410 New Zealand; ^2^ Institute of Environmental Science and Research Christchurch 8041 New Zealand; ^3^ Scion, NZ Forest Research Institute Ltd Rotorua 3010 New Zealand; ^4^ Department of Biochemistry, Genetics and Microbiology, Forestry and Agricultural Biotechnology Institute (FABI) University of Pretoria Pretoria South Africa; ^5^ NSW Department of Primary Industries Orange Agricultural Institute Australia

**Keywords:** aneuploidy, chromosome translocation, Dothideomycete, Dothistroma needle blight, forest pathogen, transposable elements

## Abstract

Dothistroma needle blight is one of the most devastating pine tree diseases worldwide. New and emerging epidemics have been frequent over the last 25 years, particularly in the Northern Hemisphere, where they are in part associated with changing weather patterns. One of the main Dothistroma needle blight pathogens, *Dothistroma septosporum*, has a global distribution but most molecular plant pathology research has been confined to Southern Hemisphere populations that have limited genetic diversity. Extensive genomic and transcriptomic data are available for a *D. septosporum* reference strain from New Zealand, where an introduced clonal population of the pathogen predominates. Due to the global importance of this pathogen, we determined whether the genome of this reference strain is representative of the species worldwide by sequencing the genomes of 18 strains sampled globally from different pine hosts. Genomic polymorphism shows substantial variation within the species, clustered into two distinct groups of strains with centres of diversity in Central and South America. A reciprocal chromosome translocation uniquely identifies the New Zealand strains. Globally, strains differ in their production of the virulence factor dothistromin, with extremely high production levels in strain ALP3 from Germany. Comparisons with the New Zealand reference revealed that several strains are aneuploids; for example, ALP3 has duplications of three chromosomes. Increased gene copy numbers therefore appear to contribute to increased production of dothistromin, emphasizing that studies of population structure are a necessary adjunct to functional analyses of genetic polymorphisms to identify the molecular basis of virulence in this important forest pathogen.

## Introduction

Dothistroma needle blight is one of the major diseases of pine trees in the world. Until the 1990s, Dothistroma needle blight was mainly known for its devastating effects on pine health in plantation forests in the Southern Hemisphere, especially in New Zealand, Australia and Africa (Drenkhan *et al*., 2016). But severe disease epidemics are now a global phenomenon, with widespread death of native and plantation pines due to *Dothistroma septosporum*, particularly in Canada and Europe (Fraser *et al*., 2016; Woods *et al*., 2005). The increased incidence and severity of Dothistroma needle blight has been associated with changes in climate, notably changing precipitation patterns (Woods *et al*., 2016). A global community effort to document and study this disease (Bradshaw, 2016) led to a species and nomenclature revision of pathogens causing the disease (Barnes *et al*., 2004), increased awareness of Dothistroma needle blight and in‐depth studies of the pathogens, their hosts and effects of the environment on disease expression (Drenkhan *et al*., 2016; Fraser *et al*., 2016; Woods *et al*., 2016).

In recent years, reference genome sequences have been obtained for many fungal pathogens of forest trees, providing a rich resource for exploring the molecular basis of pathogenicity and virulence (Dhillon *et al*., 2015; van der Nest *et al*., 2014; Olson *et al*., 2012). The genome of a New Zealand strain of *Dothistroma septosporum* was sequenced, assembled to chromosome level and currently acts as the reference genome for this species (de Wit *et al*., 2012). The genome revealed signatures of host adaptation compared to its sister species, the tomato pathogen *Cladosporium fulvum* (de Wit *et al*., 2012) and enabled comparative studies with a broader range of fungal pathogens in the Dothideomycetes (Ohm *et al*., 2012). The availability of a *D. septosporum* reference also enabled a genome‐wide study of gene expression during several temporal stages of pine needle invasion (Bradshaw *et al*., 2016), as well as more targeted studies of the production and regulation of the virulence factor dothistromin (Bradshaw *et al*., 2013, 2016; Chettri *et al*., 2013; 2018; Kabir *et al*., 2015).

Globally, populations of *D. septosporum *differ in their genetic diversity. The New Zealand *D. septosporum* strain representing the genome reference was from a clonal population (Barnes *et al*., 2014; de Wit *et al*., 2012), probably stemming from a limited introduction of the pathogen into New Zealand in the 1960s, followed by strict quarantine procedures that prevented further incursions (Barnes *et al*., 2014). In contrast, populations of *D. septosporum* from many Northern Hemisphere regions, such as Canada and Europe, show high genetic diversity (Barnes *et al*., 2014; Dale *et al*., 2011; Drenkhan *et al*., 2013), although the origin of the species remains obscure. At present, molecular knowledge of *D. septosporum* strains other than the New Zealand reference is mainly limited to mating type genes, genetic markers used for population studies (Barnes *et al*., 2014; Drenkhan *et al*., 2013; Groenewald *et al*., 2007; Mullett *et al*., 2015) and some genes associated with secondary metabolism (Ozturk *et al*., 2017). Phenotypic variation between global strains is also largely undocumented, although *D. septosporum* strains are known to differ considerably in their levels of dothistromin production, with strains from the German Alps producing significantly more of the virulence factor dothistromin than New Zealand strains (Bradshaw *et al*., 2000).

In this study, we obtained genome sequences from a diverse set of 18 strains of *D. septosporum* sampled from 15 countries. By comparing these genomes, we aimed to determine the extent to which the reference genome from the New Zealand strain is representative of *D. septosporum* globally, by comparing it to strains collected from different countries and hosts. We also aimed to look for genomic traits associated with high levels of dothistromin production.

## Results

### Sequencing, mapping and assembly of 18 *D. septosporum* genomes

Eighteen strains of *D. septosporum,* collected over a 50‐year period, were selected for this study (Table 1). Genome sequencing produced ~259 million reads (64.7 Gb of data) evenly distributed between the 18 *D. septosporum* isolates (Table 2). Paired‐end reads from 15 of the genomes mapped to the *D. septosporum* NZE10 reference genome at mapping percentages ranging from 81%–93%. However, reads from two Guatemalan (GUA1, GUA2) strains and one strain from Greece (GRE1) showed lower read mapping percentages and comparatively lower coverage (Table 2). In these three genomes, as well as that of ALP3, scaffolds that did not align to the reference genome contained gene predictions that mapped most closely to the genome of a cellulolytic *Paenibacillus* sp. strain MAEPY1 (Chua *et al*., 2014) and included 0.66% of all ALP3 reads, 2.4% of GRE1, 35% of GUA1, and 0.68% of GUA2. The high number of *Paenibacillus* sp. reads in the GUA1 sample was concordant with the lowest alignment of reads to the *D. septosporum* reference genome (Table 2). In our experience, despite meticulous sterile technique, bacteria are often associated with the growth of *D. septosporum* in culture. Our hypothesis is that the *Paenibacillus* reads may have originated from a bacterial endophyte living in, or in very close association with, the fungus.

**Table 1:** Origins of *Dothistroma septosporum* strains and dothistromin levels.

Strain ID	CMW number†	Dothistromin ng/mg DW mycelium mean ± SD	Country of origin	Region (locality if known)	*Pinus* host	Year collected	Collected by
ALP3	13122	77.84 ± 12.27	Germany	Bavarian Alps	*P. mugo*	1996	L Pehl
AUS4	15843	2.03 ± 0.70	Austria	Lower Austria (Hollenstein)	*P. sylvestris*	2004	T Kirisits
BHU1	23429	0.99 ± 0.38	Bhutan	Yusipang, Thimphu dzongkhag	*P. radiata*	2005	T Kirisits, MJ Wingfield
CAN3	14823	10.37 ± 0.60	Canada	British Columbia (Goldstream River)	*P. contorta v. latifolia*	1997	D Morrison
CHI17	10798	0.26 ± 0.06	Chile	Biobio (Canteras)	*P. radiata*	2001	MJ Wingfield
COLN	37193	8.14 ± 0.91	Colombia	Northern zone (Sonora EST‐1‐128; Armenia, Quindio)	*P. elliottii x taeda*	2011	C Rodas
COLS	37194	0.33 ± 0.10	Colombia	Southern Colombia (Don Miguel Lote 54; Popayan, Cauca)	*P. kesiya*	2011	C Rodas
DEN1	40004	1.43 ± 0.62	Denmark	Copenhagen (Hørsholm)	*P. aristata*	2013	IM Thomsen
ECU13	10211	7.49 ± 0.52	Ecuador	Lasso Highlands	*P. radiata*	2001	MJ Wingfield
GRE1	37965	0.60 ± 0.19	Greece	Northern Greece	*P. brutia or P. nigra*	2012	P Tsopelas
GUA1	44207	12.16 ± 1.38	Guatemala	Central Highlands (Sierra de Chuacus)	*P. tecunumanii*	1983	not known
GUA2	38941	16.85 ± 2.84	Guatemala	Jalapa (Mataquescuintla, Finca La Soledad)	*P. oocarpa*	2012	I Barnes
RUS1	44656	5.96 ± 0.67	Russia	St. Petersburg (Park Sosnovka)	*P. sylvestris*	2013	R Drenkhan, DL Musolin
SAF4	11305	21.58 ± 2.73	South Africa	Limpopo (Dodington Farm/Tzaneen)	*P. radiata*	2002	I Barnes
SLV1	13124	6.60 ± 1.04	Slovakia	Modrý Kameň	*P. sylvestris*	1996	L Pehl
USA12	14822	0.53 ± 0.03	USA	Oregon (Bandon)	*P. ponderosa*	1983	G Peterson
NZE10	11707	10.54 ± 0.26	New Zealand	West Coast	*P. radiata*	2005	B Doherty
NZE2	NZFS4520		New Zealand	Central North Island (Tongariro)	*P. ponderosa*	1965	JW Gilmour
NZE8	MU_NZE8		New Zealand	Bay of Plenty (Mt Maunganui)	*P. radiata*	2004	K Dobbie
AUST6*	13559		Australia	New South Wales	*P. radiata*	2003	A Carnegie

SD, standard deviation.

* Aust6 (Australian) isolate only used for PCR verification of the chromosome 5:13 translocation.

^†^ Commonwealth Mycological Institute Culture collection number (CMW), NZ forest service (NZFS) or Massey University (MU) number.

**Table 2:** *Dothistroma septosporum* genome statistics and polymorphisms.

Strain*		Reads**	Aligned to reference (%)	Coverage (fold)	SNPs†	Mean SNPs per gene (exons)	Number of deleted genes	Transposons	Assemblies‡	N50 (kb)	L50	GC content (%)	Genome fraction (%)	Total length (Mb)
		Trimmed reads			Total SNPs			Transposable Elements (% of genome)	Number of scaffolds					
NZE10	n/a		n/a	n/a	n/a	n/a	n/a	3.30	20	2596	5	53.1	100	30.2
ALP3	13195033		88.35	76	118301	4.77	69	2.59	635	665	18	53.1	95.9	30.7
AUS4	12994357		89.58	80	119648	4.78	72	3.64	174	790	13	53.0	96.0	30.3
BHU1	12911600		84.81	76	133928	5.68	67	4.83	605	315	29	53.1	95.6	31.1
CAN3	12813276		89.28	78	152609	6.42	91	2.50	456	835	11	53.2	95.3	30.1
CHI17	12336209		90.69	74	93610	3.59	66	2.61	255	748	14	53.1	97.1	30.2
COLN	13117918		81.76	72	632513	29.47	278	6.71	518	419	24	52.4	86.5	31.9
COLS	11642936		81.62	67	603570	28.01	275	6.73	570	466	20	52.4	86.7	32.0
DEN1	12382868		87.66	71	120491	4.89	61	4.51	218	500	19	52.9	96.1	30.9
ECU13	12990284		91.54	77	93800	3.59	71	2.40	132	746	14	53.1	97.1	30.2
GRE1	10813586		78.78	55	117895	4.80	83	3.89	322	500	22	53.0	96.3	30.7
GUA1	13561385		37.74	36	630549	29.70	323	19.37	3498	185	55	51.4	85.5	36.8
GUA2	12746260		68.02	61	634096	29.77	298	16.64	1803	237	45	51.5	85.6	36.4
NZE2	13166142		93.42	82	3277	0.01	7	3.03	146	1040	9	53.1	99.4	30.1
NZE8	13214724		91.07	80	3040	0.01	6	3.73	372	774	12	53.0	99.4	30.3
RUS1	12742242		91.24	80	115035	4.64	71	2.17	143	1110	10	53.2	96.1	29.9
SAF4	11716036		92.14	64	119310	4.77	55	2.87	141	713	15	53.1	96.4	30.0
SLV1	11891552		91.53	70	118915	4.85	61	3.87	343	565	17	53.0	96.2	30.6
USA12	12976213		92.77	71	153684	6.47	86	3.01	296	635	14	53.0	95.5	30.2

* Reference genome strain NZE10 (de Wit *et al*., 2012); n/a, not applicable. Strains ALP3, GRE1, GUA1 and GUA2 are believed to contain an endophytic or intimately associated *Paenibacillus* sp. mutualist. Contigs with homology to *Paenibacillus* sp. strains MAEPY1 and MAEPY2 were excluded from assembly statistics in this table, but purposely retained in the NCBI accessions for completeness.

** Aligned to reference: percentage of reads that mapped to the NZE10 reference genome (http://genome.jgi.doe.gov/Dotse1/Dotse1.home.html). Coverage: the mode fold‐coverage of the genome relative to the NZE10 reference genome.

^†^ Numbers of single nucleotide polymorphisms (SNPs) compared to the NZE10 reference genome. Number of deleted genes compared to NZE10, using CNVnator and read mapping coverage data with a threshold of >90% of the gene deleted.

^‡^ Scaffold assembly from contigs using SSPACE and gapfiller. Contig assembly using SPAdes (Kmer N77). N50 is the maximum length *x* such that using scaffolds of length at least *x* accounts for at least 50% of the total assembly length. L50 is the minimum number of contigs that produce half (50%) of the bases of the assembly (i.e. the number of contigs of length at least N50).

*De novo* genome assemblies resulted in 132–635 scaffolds for most of the genomes, but a markedly higher number of scaffolds (1803–3489) for the GUA strains (Table 2). The total assembly sizes, which excluded the *Paenibacillus* sp. scaffolds, were similar to that of the NZE10 reference genome (30.2 Mb) but those of GUA1 and GUA2 strains were larger by ~6 Mb. The genomes of the Guatemalan strains also contained the largest proportion of transposable elements (16.6%–19.4%), which may help to account for their larger size. This was more than twice as much as in the Colombian strains (COLN, COLS) and about eight times as much as the strain with the lowest level of transposable elements (RUS1 from Russia) (Table 2 and S1). In most cases, there were more RNA than DNA transposon sequences in the genomes, with a few exceptions including the Denmark (DEN1) and Slovakia (SLV1) strains (Table S1).

### Genome‐wide polymorphisms and gene deletions

**Figure 1:**
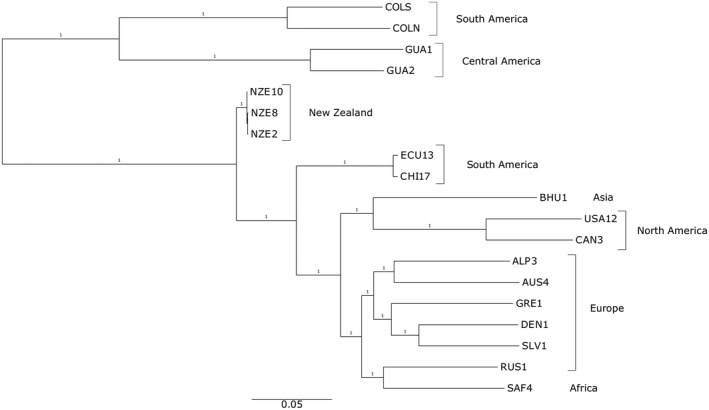
Whole‐genome single nucleotide polymorphisms (SNP) phylogeny of the sequenced strains of *Dothistroma septosporum*. Maximum likelihood phylogeny based on 5851 concatenated SNPs. The size bar represents the number of mutations per nucleotide of the proportion of the genome covered. The numbers at the nodes (all 1) are aLRT (approximate likelihood ratio test) values, indicating high confidence in the branches.

The sequences of the 18 *D. septosporum* genomes were compared to that of the NZE10 reference to determine the numbers and locations of single nucleotide polymorphisms (SNPs). As expected, New Zealand strains NZE2 and NZE8 had very few SNPs relative to NZE10 (average 0.01 SNPs per gene; Table 2), in keeping with the almost clonal nature of the New Zealand *D. septosporum* population. In striking contrast, the Guatemalan and Colombian strains had a large number of sequence differences from NZE10 (average 29 SNPs per gene) with other samples between these extremes (Table 2). A phylogeny prepared using whole‐genome SNP information highlights the genetic distance of the Colombian and Guatemalan strains from the rest of the samples, but also shows that they are distinct from one another (Fig. 1). In general, the SNP phylogeny showed that strains are grouped by geographic origin. However, there are exceptions: The South African strain (SAF4) was more similar to the Russian strain (RUS1) than any others; and the strain from Bhutan in Asia (BHU1) clustered with the North American strains (CAN3, USA12).

To estimate the numbers of genes that were absent in the re‐sequenced genomes compared to NZE10, the statistical package CNVnator was run using read mapping coverage data. Similar to the SNP results, the predicted levels of gene absence, compared to NZE10, were lowest in the other New Zealand strains and highest in the Guatemalan and Colombian strains (Table 2). In total, almost 600 of 12 580 genes (4.8%) appeared to be deleted in at least one strain across all 18 re‐sequenced genomes. Based on an earlier transcriptome study of gene expression of the NZE10 strain *in planta *(Bradshaw *et al*., 2016), 77% of these deleted genes had very low expression levels in the reference genome (<20 reads/million/kb; Table S2). Some of the genes predicted to be absent in Colombian strains, and to some extent in Guatemalan strains, were highly expressed by NZE10 *in planta* (Table S2). But many of those highly expressed genes had no known function or were predicted to encode oxidoreductases or methyltransferases that are likely to have redundant functions due to other similar genes in the genome (Table S2).

Because the NZE10 reference genome is assembled to chromosome level (de Wit *et al*., 2012), the relative rates of estimated deletions and SNPs per chromosome were determined amongst the re‐sequenced strains (Table 3). Both mean numbers of SNPs per kb of exon and gene deletions per length of chromosome were lowest for chromosome 1 and highest for chromosome 14. This reflects the overall hypervariability of chromosome 14, even though its repeat content is relatively low (Table 3). This hypervariability was also seen in the read copy number variation plots per chromosome, with many large deletions observed across chromosome 14, particularly in the Colombian and Guatemalan strains (Fig. S1). Of 61 deleted genes on chromosome 14, only eight had functional predictions, and only five were expressed above a threshold of 20 reads/million/kb by NZE10 *in planta*. The more highly expressed genes included predicted DNA binding, membrane‐associated and phosphate ion transporter genes (Table S3).

**Table 3:** Single nucleotide polymorphisms (SNPs) and gene deletions by NZE10 chromosome.

NZE10 chromosome	Length bp*	Mean exon SNPs†	Mean SNPs/kb exon	% repeats*	Number of genes	Number genes deleted	% genes deleted	Number del/length‡ × 10^6^	Times deleted total‡	Total del/length × 10^6^
1	5111597	18761	6.7	1.42	2148	32	1.5	6.3	99	19.4
2	3306877	12953	7.1	1.26	1382	38	2.7	11.5	147	44.5
3	2752214	11140	8.2	4.68	1079	43	4.0	15.6	233	84.7
4	2620707	10707	8.4	3.16	1060	40	3.8	15.3	154	58.8
5	2595548	11382	8.8	2.48	1142	59	5.2	22.7	170	65.5
6	2187679	8897	8.2	5.35	925	51	5.5	23.3	169	77.3
7	2106062	8115	7.7	4.82	846	40	4.7	19.0	95	45.1
8	1927254	8395	8.3	1.97	745	51	6.8	26.5	178	92.4
9	1757826	7482	8.2	3.94	861	29	3.4	16.5	78	44.4
10	1635767	7120	8.9	3.93	681	30	4.4	18.3	97	59.3
11	1558309	6499	8.5	4.71	679	42	6.2	27.0	120	77.0
12	1256034	5373	9.2	5.45	509	47	9.2	37.4	152	121.0
13	962570	4288	9.5	2.90	418	32	7.7	33.2	131	136.1
14	407968	2046	13.4	1.79	164	61	37.2	149.5	209	512.3

* Data from de Wit *et al*. (2012).

^†^ SNPs compared to NZE10.

^‡^ Number of deleted genes per chromosome, compared to NZE10 and normalized by scaffold length. Times deleted total is number of gene deletions in all 18 genomes.

### A reciprocal translocation in the NZE10 reference genome

**Figure 2:**
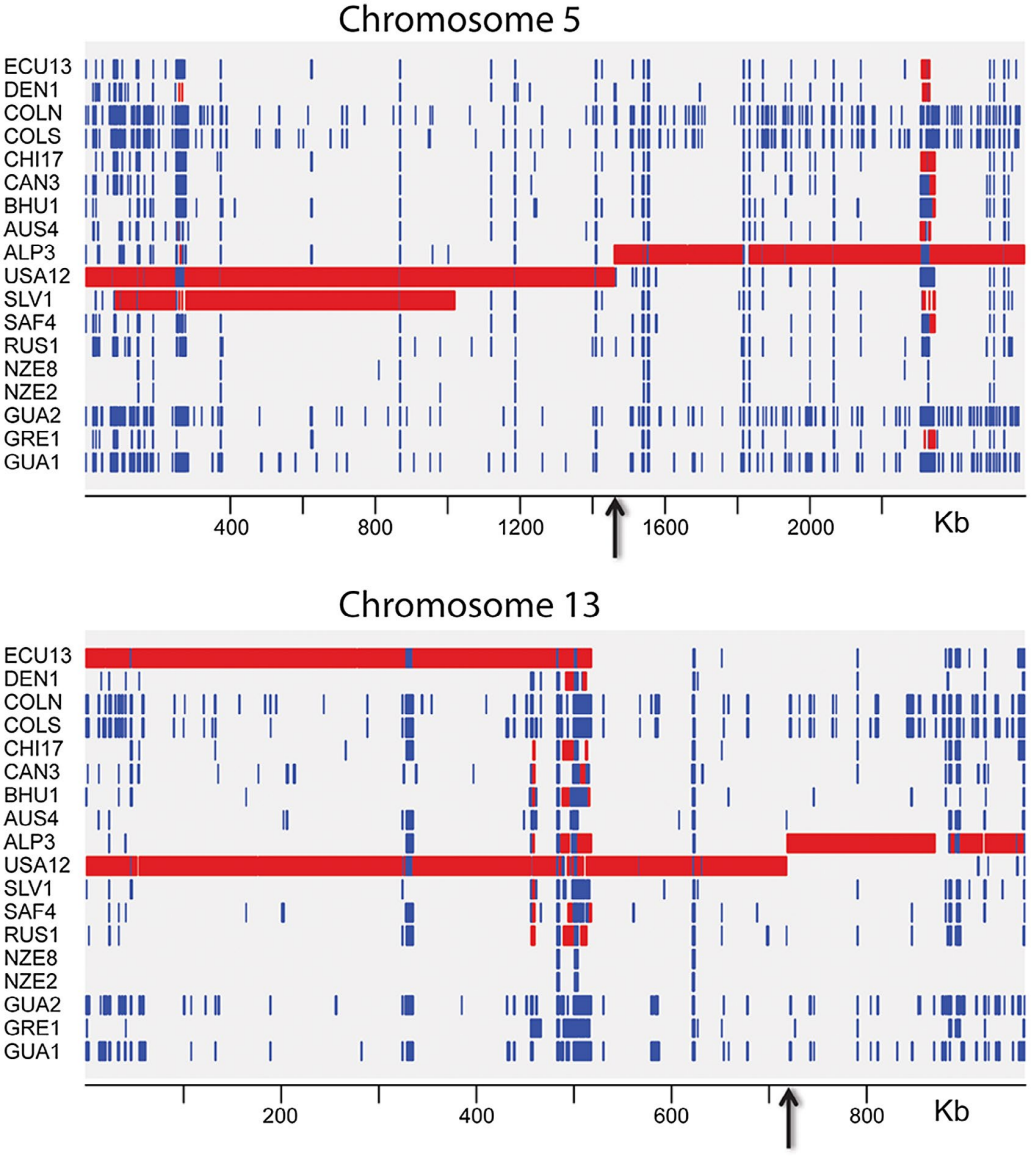
Predicted duplications and deletions across *Dothistroma septosporum* chromosomes 5 and 13. Regions of duplications (red) and deletions (blue) predicted on the basis of read mapping coverage using the copy number variant tool CNVnator. The central breaks in predicted duplication coverage in the ALP3 and USA12 strains occur precisely at the translocation breakpoints (indicated with black arrows). Verification with quantitative Polymerase Chain Reaction (qPCR) (Table 4) suggested that the ALP3 strain has a second copy of the small (1.4 Mb) 5:13 ‘translocation’ chromosome.

**Figure 3:**
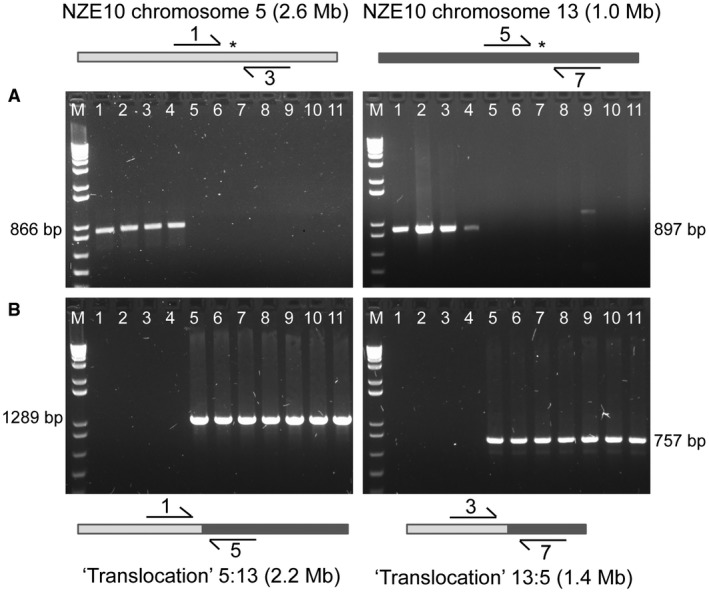
Polymerase Chain Reaction (PCR) confirmation of a chromosome 5:13 translocation in the NZE10 reference genome. PCR amplification products from *Dothistroma septosporum* genomic DNA, with different combinations of primers (indicated by numbers on arrows) that flank the translocation breakpoints. Primer sequences are shown in Table S7. Lanes 1–4 contain PCR products from New Zealand and Australian strains (1 NZE10, 2 NZE2, 3 NZE8, 4 AUST6); lanes 5–11 are from other regions of the world (5 USA12, 6 ALP3, 7 SLV1, 8 COLN, 9 GUA2, 10 SAF4, 11 ECU13); M is a 1 kb ladder size marker and sizes of the main amplicons are indicated. Diagrams above and below the panels (not drawn to scale) show the combinations of primers used. (A) The top combinations amplify within chromosome 5 or 13 of the NZE10 reference genome. (B) the bottom combinations amplify across the junction of a ‘translocation’ of chromosomes 5 and 13. Note, that the ‘translocation’ as shown at the bottom is actually the normal situation seen in most global strains; a translocation most likely occurred in a progenitor of the NZE and Australian isolates to produce the arrangement seen at the top.

During analysis of paired‐end read and contig mapping to the *D. septosporum* NZE10 reference genome, a reciprocal translocation was discovered between chromosomes 5 and 13 in NZE10 compared to strains from other continents. Three initial lines of evidence led to this discovery. Firstly, sharp transitions in read coverage were seen at reciprocal positions for reads from the USA12 and ALP3 strains that mapped within chromosomes 5 and 13, coinciding exactly with the 5:13 translocation breakpoint in NZE10 (Fig. 2). Secondly, single contigs from many of the newly assembled genomes aligned to both chromosomes 5 and 13 of the NZE10 reference genome (Fig. S2a). Thirdly, paired‐end reads from all the genomes, except others from New Zealand, failed to map together over the region of the translocation junction (Fig. S2b). Polymerase Chain Reaction (PCR) amplification across the putative translocation junctions, and sequence analysis of the PCR products, confirmed the presence of the translocation in an additional Australian *D. septosporum* isolate whose genome had not been sequenced, as well as in the sequenced New Zealand isolates, and its absence from the genomes of all isolates from other parts of the world (Figs 3, S3).

In the translocation, fragments of chromosomes 5 (2.6 Mb) and 13 (1 Mb) (as in the NZE reference genome) form a 2.2 Mb chromosome (with the two long fragments) and a 1.4 Mb chromosome (with the two short fragments) in the global strains (Figs. 3, S3a and S3b). The breakpoints in chromosomes 5 and 13 contain a common 7 bp repetitive sequence (GCGCGGT) and occur in intergenic regions where there is no evidence of larger repeats or transposable elements. Genes flanking the breakpoints in both chromosomes are divergently transcribed and in each case include one gene that has very low expression and one with moderate expression levels *in planta* in NZE10 (Fig. S3c and S3d). The low‐expressed gene on chromosome 13 (protein ID 48707) is a predicted alpha‐beta hydrolase gene whilst the other three genes flanking the breakpoints had no functional predictions from Gene Ontology (GO) or KOG analysis.

### Dothistromin toxin and dothistromin genes

Dothistromin is a known virulence factor in Dothistroma needle blight (Kabir *et al*., 2015) and different isolates of *D. septosporum* vary greatly in the levels of dothistromin they produce in culture (Bradshaw *et al*., 2000). Thus, we determined levels of dothistromin production in the 18 strains and looked for associations of genomic features with dothistromin levels. Table 1 shows that strain ALP3 produced over seven times more dothistromin than the NZE10 reference strain, almost four times more than the next‐highest producer, SAF4, and over 30 times more than the strain from Austria (AUS4) which grouped most closely with ALP3 in the phylogeny (Fig. 1). This very high level of dothistromin production by ALP3 is similar to that shown almost two decades ago (Bradshaw *et al*., 2000), albeit grown under different conditions and assayed using a different method. This suggests that years of storage (cryopreservation and storage of mycelial agar plugs in dH_2_O at 4 °C) has not resulted in attenuation or a decrease in dothistromin levels.

To identify potential genetic origins for differences in dothistromin levels, we first investigated the well‐characterized dothistromin genes. Twenty biosynthetic and regulatory genes involved in dothistromin biosynthesis are spread across six loci on chromosome 12 (Chettri *et al*., 2013). All dothistromin genes were present in all genomes and appeared to be conserved, based on a low proportion of non‐synonymous SNPs and low dN/dS values indicative of negative selection (Table S4). Because previous studies with NZE10 showed that AflR is a key pathway regulator of dothistromin genes (Chettri *et al*., 2013), we then compared *AflR* sequences across the strains. AflR is highly conserved, with only six amino acid polymorphisms over the protein length of 479 amino acids amongst all the *D. septosporum* genomes (Fig. S4). Of these, five sites were also polymorphic between AflR proteins from *D. septosporum, Cladosporium fulvum, Aspergillus parasiticus* and *Aspergillus nidulans*, whilst the sixth site (N349K) was conserved (as N) between those species (Chettri *et al*., 2013) but varied only in the high dothistromin *D. septosporum* ALP3 strain. However the ALP3 N349K polymorphism did not occur in a region of AflR known to be of functional importance (Fig. S4). Secondary structure predictions using HHpred (Hildebrand *et al*., 2009), suggested similar structures for NZE10 and ALP3 AflR proteins over the region of the polymorphism (Fig. S5), making mutation of AflR an unlikely cause of the high dothistromin levels in ALP3. Due to regulatory restrictions on import of *D. septosporum* into New Zealand, we were not able to compare expression levels of dothistromin genes between the strains.

The effect of the structure of chromosome 12 on dothistromin production was analysed. In chromosome 12 of NZE10 a large (~100 kb) transposon‐rich repeat region occurs just upstream of the dothistromin *OrdB* gene in dothistromin gene cluster locus 5. This is seen by the large blue ‘deletion’ blocks in the copy number variant analysis plot for chromosome 12 (Fig. S1). Many of the strains had large deletions in this region that included the loss of 10 genes adjacent to the repeat area in NZE10 (Tables S1 and S5). From available RNA‐seq data (Bradshaw *et al*., 2016), none of these ten genes were highly expressed in NZE10, with maximum reads/million/kb values of only 73 in culture and 76 *in planta* for a kinase gene (protein ID 57306) (Table S5).

Amongst the strains from Colombia, COLN had considerably higher levels of dothistromin production (24‐fold higher) than COLS (Table 1). Read copy number analysis (Fig. S1 and Table S6) suggested an approximately 500 kb duplication of the first part of chromosome 12 in the COLN strain that encompassed the locus containing the dothistromin *DotB* and *DotC* genes, whilst the COLS strain was not predicted to have this duplication. However, repeated attempts to verify duplication of *DotB* and *DotC* genes in COLN by quantitative PCR (qPCR) gave ambiguous results.

### Evidence for aneuploidy

CNVnator analysis of read mapping coverage data predicted that the ALP3 strain also has duplicated regions in its genome, although not on chromosome 12 where the characterized dothistromin genes are located (Table S6). These, and other duplications as shown in Table 4, were confirmed by qPCR copy number analysis of selected genes, with the exception of a predicted duplication of chromosome 13 (long arm) in the USA12 strain that was not supported by qPCR. ALP3 appears to have a duplication of the 1.4 Mb ‘T13:5’ translocation chromosome shown in Fig. 3, with duplication of genes on the ‘5 short’ and ‘13 short’ chromosome fragments (Table 4). Furthermore, the borders of duplicated regions that were predicted by CNVnator finished precisely at the experimentally verified translocation breakpoints (Table S6 and Fig. S3). The 1.4 Mb T13:5 chromosome, which is duplicated only in the ALP3 strain, contains ~600 genes, of which ~120 are predicted to be involved in metal‐ion binding, DNA binding, transmembrane transport, signalling, translation, oxidoreductase or methyltransferase activity. It is feasible that a combination of extra copies of some of these genes could account for the increased dothistromin production seen in the ALP3 strain.

**Table 4:** Copy number variant validation by quantitative Polymerase Chain Reaction (qPCR)

Chromosome*	Location	Gene†	Gene copy number estimates‡ for four strains			
			ALP3	SAF4	SLV1	USA12
5 long	795197	Ds71757	0.92 ± 0.04	1.20 ± 0.15	3.20 ± 0.44*	2.33 ± 0.43*
	764916	Ds71743	0.87 ± 0.03	1.16 ± 0.17	2.91 ± 0.79*	1.84 ± 0.02*
5 short	2546495	Ds72297	2.30 ± 0.16*	1.06 ± 0.05	1.06 ± 0.03	1.07 ± 0.17
	2080702	Ds72172	2.35 ± 0.04*	0.88 ± 0.16	0.86 ± 0.28	0.99 ± 0.15
	1505739	Ds72010	1.92 ± 0.07*	1.06 ± 0.06	0.89 ± 0.03	0.80 ± 0.12
9	914042	Ds157678	1.22 ± 0.02*	2.80 ± 0.72*	1.03 ± 0.36	2.12 ± 0.19*
11	1319989	Ds75320	2.66 ± 0.02*	4.38 ± 0.26*	1.10 ± 0.13	2.30 ± 0.03*
13 long	69203	Ds161036	1.12 ± 0.04	1.18 ± 0.17	1.08 ± 0.10	1.15 ± 0.09
	161624	Ds75737	0.88 ± 0.02	1.02 ± 0.15	0.81 ± 0.06	0.84 ± 0.40
13 short	749433	Ds29553	1.90 ± 0.08*	1.02 ± 0.03	1.03 ± 0.10	1.00 ± 0.05
	828406	Ds75914	2.07 ± 0.08*	1.05 ± 0.15	1.08 ± 0.07	1.35 ± 0.16
14	116999	Ds75967	2.09 ± 0.00*	2.21 ± 0.15*	1.15 ± 0.03	1.89 ± 0.02*

* Chromosome of *Dothistroma septosporum* NZE10 to which reads from the other strains were mapped.

^†^ Target genes for qPCR, located in regions predicted by CNVnator to be duplicated (Table S6).

^‡^ Mean ± standard deviation (SD) gene copy number estimate determined by qPCR compared to the single copy gene *AflR* and normalized to the genome reference strain NZE10. Significantly higher target/reference ratios compared to NZE10 are indicated with an asterisk (*P* < 0.01). Dark grey shading indicates CNVnator prediction of entire chromosome duplication (or long/short section of chromosome 5 or 13); light grey shading indicates partial duplication (see Table S6).

ALP3 was also predicted to have duplications of entire chromosomes 11 and 14 (Table S6). Some genes with potential roles in boosting dothistromin production are present on chromosome 11, although these duplications were shared with two other strains: USA12 (lowest dothistromin producer) and SAF4 (high producer). Amongst these genes, Ds75171 is an orthologue of *A. nidulans* AN5169 (Boi1), a phospholipid binding protein gene recently implicated in regulation of secondary metabolism (Pfannenstiel *et al*., 2017). Another gene present on chromosome 11 that could facilitate high levels of dothistromin production is a putative major facilitator superfamily (MFS) transporter gene Ds75255. The predicted Ds75255 gene product was a reciprocal best BLAST hit to *Botrytis cinerea* Bcmfs1, a protein shown to confer resistance to a range of toxic compounds including the polyketide toxin cercosporin (Hayashi *et al*., 2002).

## Discussion

### Variation amongst the genomes

Despite the small sample size of 19 genomes, the whole‐genome phylogeny (Fig. 1) reveals similar features to the previous global *D. septosporum* population structure study of 458 isolates from 14 countries (Barnes *et al*., 2014). The European strains (ALP3, AUS4, GRE1, DEN1 and SLV1) formed a long‐branched clade and were distinct from North American (USA12, CAN3) and Asian (BHU1) strains. South American (ECU13, CHI17) and New Zealand (NZE) strains, all collected from *P. radiata*, formed two clades each with short terminal branches consistent with the clonal structure of those populations (Barnes *et al*., 2014). Only one African strain was included in our study (SAF4) and this grouped with the neotype strain from Russia (RUS1; Barnes *et al.*, 2016), although with long branch lengths indicative of divergence.

The strains from Guatemala (GUA1, GUA2) and Colombia (COLN, COLS) were distinct from all other strains in this study and from each other. The four GUA and COL genomes showed higher levels of SNPs, deleted genes and transposable elements, particularly RNA transposons, than most of the other strains. They formed a separate clade in the whole‐genome phylogeny, suggesting that they may represent new lineages of *D. septosporum*. However, each of these four strains was isolated from a different *Pinus* species compared to all other strains and this alone could potentially account for the differences found. Host specificity studies and further population analyses are required to determine the evolutionary history of these lineages. As transposable elements are known to be involved in genome evolution and the adaptation of fungal pathogens (Faino *et al*., 2016; Moller and Stukenbrock, 2017), these might be associated with genomic deletions and diversification of the GUA and COL isolates. As the genome assemblies of the isolates are improved in the future, the distribution and expansion of transposable elements in these isolates can be further investigated.

DNA sequencing revealed that the genomes of the GUA strains, along with those of GRE1 and ALP3, contained bacterial DNA putatively identified as a species of *Paenibacillus*. Our hypothesis that the *Paenibacillus* reads may have originated from a bacterial endophyte living in, or closely associated with, the fungus is supported by other studies. *Paenibacillus* spp. are associated with mycorrhizal fungi as ‘helper bacteria’ in roots of *Pinus sylvestris* and other species (Aspray *et al*., 2006; Li *et al*., 2008), can stimulate growth of mycorrhizal fungi in culture (Hildebrandt *et al*., 2006) and have been shown to live intracellularly in *Laccaria bicolor* (Bertaux *et al*., 2003). *Paenibacillus* sp. reads were also recently reported as ‘bacterial contaminates’ in genome sequences of two Australian strains of the broad‐host‐range pathogen *Phytophthora cinnamomi* (Longmuir *et al*., 2017). The association of *Paenibacillus* sp. with *D. septosporum* needs to be investigated further.

### The Australasian chromosome 5:13 translocation

A translocation involving chromosomes 5 and 13 was discovered by cross‐mapping of paired‐end reads, and of assembled contigs, to both chromosomes. In the NZE10 reference strain, chromosomes 5 and 13 are well assembled with telomeres at both ends. PCR amplification across the translocation junction showed that all of the New Zealand and Australian strains had the same chromosome arrangement as NZE10 whilst strains from the rest of the world have the ‘translocation’. Given their clonal origin, it is most likely that the translocation occurred in a progenitor of the Australasian strains (Barnes *et al*., 2014; Hirst *et al*., 1999), and that the global strains have the normal chromosome arrangement. Further support for this hypothesis can be seen in a comparison with the Dothideomycete banana pathogen *Pseudocercospora fijiensis*; a mesosynteny plot showed that *D. septosporum* chromosomes 5 and 13 are both split, with the different sections corresponding to large scaffolds 3 and 8 from *P. fijiensis *(Isaza *et al*., 2016). This suggests that *P. fijiensis* has the same arrangement of these chromosomes as most of the global *D. septosporum* strains.

Chromosome evolution in Dothideomycete fungi is associated with high levels of mesosynteny, in which intrachromosomal rearrangements due to inversions result in different orders and orientations of genes between homologous chromosomes in different species (Hane *et al*., 2011). In contrast, interchromosomal rearrangements like translocations are relatively rare in this class of fungi (Hane *et al*., 2011) although translocations were reported for *P. fijiensis *(Isaza *et al*., 2016). Some chromosome rearrangements have been associated with virulence, such as in the corn pathogen *Cochliobolus heterostrophus. *Race T strains of this pathogen produce the T‐toxin virulence factor, for which two complex loci near the breakpoints of a reciprocal translocation (*Tox1A* and *Tox1B*) contain T‐toxin biosynthetic genes (Kodama *et al*., 1999; Turgeon and Baker, 2007). In fungi outside of the Dothideomycetes, extensive intra‐ and interchromosomal rearrangements occurred between strains of the broad‐host‐range asexual pathogen *Verticillium dahliae*, leading to the suggestion that chromosome reshuffling can provide a mechanism for selection in asexual pathogens (de Jonge *et al*., 2013). Transposable elements have been implicated in translocation events, such as interchromosomal transfer of the *AVR‐Pita* effector gene in the rice blast fungus *Magnaporthe grisea* (Chuma *et al*., 2011).

The discovery that the NZE10 reference genome has a chromosome translocation shows that the genome structure differs from that of most global strains. However, there was no evidence for any phenotypic effects of the *D. septosporum* 5:13 translocation, and to the best of our knowledge none of the genome is missing. Hence the genomic information is just reshuffled in NZE10 and the other Australasian strains. The chromosome‐level assembly of the NZE10 genome (de Wit *et al*., 2012) has not yet been achieved for any other *D. septosporum* strain. Thus, we advocate keeping the current NZE10 genome sequence as the species reference for continuity.

### Is chromosome 14 dispensable?

Analysis of *D. septosporum* chromosome 14 copy number variation revealed considerable plasticity in its structure, with higher rates of gene deletions, duplications and also predicted SNPs, compared to chromosomes 1‐13. Many fungal pathogens, such as *Zymoseptoria tritici* (as *Mycosphaerella graminicola*) (Goodwin *et al*., 2011), *Fusarium* *oxysporum* (Ma *et al*., 2010) and *Alternaria* *alternata *(Hatta *et al*., 2002) have dispensable chromosomes (also known as accessory or supernumerary chromosomes). These chromosomes tend to be small in size, repeat‐rich, gene‐sparse and often contain virulence‐associated genes (Moller and Stukenbrock, 2017). Dispensable chromosomes also tend to evolve at different rates; in *Z. tritici* the non‐synonymous substitution rate was three times higher in dispensable than essential chromosomes (Stukenbrock *et al*., 2010) and their rapid evolution has been associated with chromosomal rearrangements involving breakage, fusion and insertion events (Croll *et al*., 2013). However, we found no evidence that *D. septosporum* chromosome 14 is dispensable. Amongst the strains studied here, all had at least 103 of 164 genes that are associated with chromosome 14 in NZE10. Furthermore, the repeat content of chromosome 14 in NZE10 is the third lowest (1.8%) of any of the chromosomes (de Wit *et al*., 2012). However, a larger set of *D. septosporum* strains should be studied to confirm this observation.

### A genetic basis for variation in dothistromin levels?

The ALP3 strain showed high levels of dothistromin production in culture compared to all other strains, consistent with earlier work (Bradshaw *et al*., 2000). Whether these high levels are indicative of high levels of dothistromin in the forest situation is not known. Likewise, it is not known if the ALP3 strain has increased virulence compared to lower dothistromin producers, although this might be expected as dothistromin is a known virulence factor (Kabir *et al*., 2015). Amongst the *D. septosporum* strains sequenced, ALP3 was the only one isolated from *Pinus mugo*, a very susceptible host.

The ALP3 genome appears to have duplications of the ‘translocated’ 13:5 1.4 Mb chromosome as well as chromosomes 11 and 14, with higher read mapping rates and higher copy numbers confirmed by qPCR of genes located in those regions. Although aneuploidy is typically unstable in fungi grown in laboratory conditions, it is common in wild isolates of fungi and has been associated with increased fitness under extreme or stressful conditions (Berman, 2016). In a study of 38 isogenic stable aneuploid lines of *Saccharomyces cerevisiae*, whilst most of the aneuploids grew more slowly under ‘optimal’ lab conditions, some showed improved fitness over euploid strains when grown in sub‐optimal conditions with chemical or environmental stressors (Pavelka *et al*., 2010). Similarly, in clinical isolates of *Candida albicans*, aneuploidy was associated with tolerance to the antifungal drug fluconazole (Selmecki *et al*., 2009) and it has been proposed that aneuploidy can be used by fungi as an adaptation to stress (Berman, 2016).

It is possible that the aneuploidy in ALP3 contributed to its high dothistromin levels due to increased dosage of one or more genes. Aneuploidy is known to lead to increased levels of gene products in other fungi. In *Saccharomyces cerevisiae*, analysis of aneuploids revealed concordance of gene dosage with protein expression levels (Pavelka *et al*., 2010). In *Candida albicans* extra copies of a transcriptional activator gene for a transporter was implicated in azole drug resistance (Selmecki *et al*., 2009). Strains of *Penicillium chrysogenum* that produce very high levels of penicillin contain additional copies of the penicillin biosynthetic gene cluster in tandem repeats (Specht *et al*., 2014). In our study, the duplication of the 1.4 Mb 13:5 ‘translocation’ chromosome was unique to ALP3, and extra copies of genes involved in sugar transport, DNA binding, translation or metabolism present on this chromosome could have improved the efficiency of dothistromin production. However, functional studies with over‐expressed or multi‐copy genes are required to validate this.

Duplication of chromosome 11 in ALP3 (as well as in strains SAF4 and USA12) might also have increased the capacity to make dothistromin, as this chromosome contained potential regulatory genes as well as an orthologue of the *B. cinerea* Bcmfs1 multidrug MFS transporter (Hayashi *et al*., 2002). MFS transporters can provide a resistance mechanism for fungi against their own toxins by facilitating toxin secretion, and can also affect toxin production levels. An MFS transporter gene, *DotC*, clustered alongside dothistromin biosynthetic genes in *D. septosporum* was shown to have only a minor role in dothistromin secretion (Bradshaw *et al*., 2009). Thus, it is feasible that the Bcmfs1 orthologue could assist in secretion of dothistromin. This, along with increased expression from genes on the duplicated translocation chromosome, might account for the high dothistromin production by the ALP3 strain as well as that of the SAF4 strain, which had the second highest dothistromin level.

Other factors might have accounted for differences in dothistromin production. The very high dothistromin production by the ALP3 strain might be associated with a stress response related to the aneuploid condition, due to mutations in specific genes or associated with differences in epigenetic markers that are known to regulate dothistromin production (Chettri *et al*., 2018).

### Conclusions

In summary, a global survey of genome sequences of 18 *D. septosporum* strains suggested that the NZE10 New Zealand genome is a representative reference for all the strains with the possible exception of Guatemala and Colombia, which form a distinct clade. However the New Zealand and Australian strains do have a reciprocal translocation involving chromosomes 5 and 13. Some of the strains appear to have been associated with a *Paenibacillus* sp. bacterium and some appear to be aneuploids. In addition, we showed that levels of dothistromin production in culture were highly variable between the strains, but found no evidence for genetic differences in the dothistromin biosynthetic or regulatory genes that could account for this variability. Instead we suggest that gene copy number variation due to aneuploidy might influence dothistromin production levels in the very high‐producing ALP3 strain. Further studies with a larger number of strains would enable association studies that could determine the effects of specific SNPs, or of differences in expression of particular genes, with increased production of dothistromin.

## Experimental procedures

### Fungal strains, culture conditions and DNA extractions

Eighteen isolates, confirmed to be *D. septosporum* by ITS sequencing (Barnes *et al*., 2004), were chosen for this study and are shown in Table 1. For DNA extraction, actively growing *D. septosporum* were inoculated onto 2% Dothistroma sporulation medium (DSM) (Bradshaw *et al*., 2000) with 100 mg/L streptomycin (Sigma‐Aldrich, Saint Louis, USA) and incubated at 22 °C in natural light for 4 weeks. Mycelium was scraped from the surface of the media and freeze‐dried overnight. Samples were macerated and DNA was extracted using a method adapted from Goodwin *et al*. (1992). Due to strict New Zealand biosecurity regulations, genomic DNA from non‐New Zealand (NZ) strains was imported into NZ after extraction from cultures stored at the Forestry and Agricultural Biotechnology Institute (FABI), University of Pretoria, South Africa.

### DNA sequencing and read mapping

Library preparation and sequencing was carried out by the Australian Genome Research Facility Ltd. (AGRF) using an Illumina gDNA shotgun library preparation method with bead size selection. All 18 strain libraries were sequenced on one lane of an Illumina HiSeq2500 (Illumina, San Diego, CA, USA). The raw 125 bp paired‐end DNA sequence data were processed with fastq‐mcf to remove sequencing adapters and primers (Aronesty, 2011). The reads were quality trimmed to a Phred score of >20 using SolexaQA v3.1.4 (Cox *et al*., 2010), and trimmed reads of length <50 were discarded. Analysis of general quality parameters of the raw and processed data was done using FastQC v0.11.5 (Bioinformatics, 2011). Sequence data were deposited in the Sequence Read Archive (http://www.ncbi.nlm.nih.gov/sra/) under accession number SRP103141.

Paired‐end reads were mapped to the *D. septosporum* reference genome (New Zealand strain NZE10; http://genome.jgi.doe.gov/Dotse1/Dotse1.home.html) (de Wit *et al*., 2012) using Bowtie 2 v2.2.6 (Langmead and Salzberg, 2012). Genome‐wide coverage was determined using bedtools v2.19.1 (Quinlan and Hall, 2010). Gene coverage was determined using the current annotated *D. septosporum* loci (entire coding sequence, including introns) as windows in bedtools.

### *De novo* genome assemblies

The *de novo* assemblies were performed using SPAdes v3.6.1 (Bankevich *et al*., 2012) at a Kmer value of 77, after optimisation of contig lengths using Kmers ranging from 21 to 99. N50, L50 and GC content were calculated using OcculterCut v1.1 (Testa *et al*., 2016). The contigs were aligned to the NZE10 reference genome using the nucmer tool as part of the MUMmer package v3.23 and visualized with mummerplot (Kurtz *et al*., 2004). Scaffold assembly from the SPAdes contigs was done with PILON v1.22 (Walker *et al*., 2014) using *D. septosporum* NZE10 as a reference. The assemblies were deposited at https://www.ncbi.nlm.nih.gov/genome/genomes/11861 as BioProject accession number PRJNA381823. Assemblies for four of the *D. septosporum* strains contained scaffolds that matched to *Paenibacillus* sp. and did not align to the NZE10 genome. Homology between these scaffolds and reference genomes for the closest‐matching *Paenibacillus* sp. (NCBI accessions PRJNA217884 and PRJNA217885) was determined by D‐GENIES v1.2.0 (Cabanettes and Klopp, 2018).

### SNP analysis and estimations of selection

Mapped reads were analysed using freebayes v1.1.0‐46 (Garrison and Marth, 2012) with ploidy set to 1 (haploid) to detect variants between each of the 18 samples and the NZE10 reference. The resulting VCF files were annotated based on the *D. septosporum* NZE10 gene models (https://genome.jgi.doe.gov/Dotse1) using SnpEff v4.3t with default parameters, before quality filtering at Q ≥ 30 with SnpSift (Cingolani *et al*., 2012). To estimate selection from the SNP data, the ratio of non‐synonymous to synonymous nucleotide substitutions (dN/dS) was determined for each gene between each pair of the 18 genomes using codeml from the PAML v4.8 package (Yang, 2007). A phylogeny of concatenated SNPs from all of the genomes was built by aligning nucleotide sequences with MAFFT using the E‐INS‐I parameters (Katoh *et al*., 2005). Trees were then built using PhyML with default parameters for the maximum likelihood method (Guindon *et al*., 2010).

### Determination of deletions and duplications by copy number variation

The copy number variant tool CNVnator v0.3.2 (Abyzov *et al*., 2011) was run with default parameters with a window size of 50 bp on the bam files generated from mapping to the NZE10 reference genome. Using bedtools (Quinlan and Hall, 2010), coverage on the proportion of the gene models in the *D. septosporum* NZE10 annotation file that are covered by either deleted or duplicated regions was measured. A gene was classified as deleted if more than 90% of its gene region fell into a deleted region.

### *De novo* prediction of repetitive elements

The repetitive content and transposable elements of all 18 genomes were predicted using the REPET package (Flutre *et al*., 2011) as follows. The TEdenovo pipeline was used for *de novo* identification of consensus transposable element families and this was used as the input in the TEannot pipeline for the classification of all repetitive elements. The results were used to determine the amount of Type I and Type II transposable elements in the genomes.

### PCR verification of the translocation and gene copy numbers

PCR reactions to verify the chromosome 5:13 translocation were performed with an Eppendorf Gradient Mastercycler^® ^(Eppendorf, Hamburg, Germany); primer sequences are shown in Table S7. PCR reactions (10 μL) contained 5 ng of genomic DNA, 100 μM dNTPs, 1.5 mM MgCl_2_, 1.0 μL of 10X PCR buffer (10 mM Tris‐HCl, 50 mM KCl, 1.5 mM MgCl2, pH 8.0), 5 pM of each primer and 1 U of Platinum Taq DNA polymerase (Invitrogen, CA, USA). Cycling conditions included denaturation at 94 °C for 5 min, followed by 30 cycles of 94 °C for 30 s, annealing temperature (depending on primer) for 30 s, and 72 °C for 1 min/kb of amplicon, then a final extension at 72 °C for 10 min.

Copy numbers of the potentially duplicated regions were determined following the qPCR strategy outlined previously (Chettri *et al*., 2015), in which the crossing point (Ct) values of target gene and reference genes in the putative duplicated region were compared to those in the *D. septosporum* NZE10 reference strain. The values were normalized to a reference gene (*AflR*) known to be present as a single copy in both NZE10 (Chettri *et al*., 2013) and the other strains under investigation. Target genes in the potentially duplicated regions were selected based on GO and expression under *in vitro* conditions (Bradshaw *et al*., 2016). Primer sequences for the target genes are shown in Table S7 and standard curves were constructed for each primer pair using a series of fivefold dilutions to give a range from 1.28 pg to 20 ng of *D. septosporum* genomic DNA per 10 μL reaction. A qPCR was performed using a SensiFAST™ SYBR No‐ROX kit (Bioline, London, UK) on a LightCycler® 480 System (Roche) with PCR conditions of 5 s at 95 °C, then 10 s at 60 °C and 20 s at 72 °C, with an acquisition temperature of 72 °C.

### Dothistromin assays

To determine levels of dothistromin production in culture, *D. septosporum* isolates were grown in triplicate in DM broth as above, except flasks were inoculated with approximately 200 mg–300 mg of mycelium that was homogenized with a Retsch GmbH MM301 mixer mill (Haan, Germany). After 7 days of incubation, the culture filtrates containing secreted dothistromin were harvested by filtration and the dry weights of mycelium determined for each flask. Dothistromin was extracted from the filtrates with ethyl acetate containing 0.1% formic acid and quantified using HPLC (high‐performance liquid chromatography), as described previously (Chettri *et al*., 2012).

## Accession numbers

NCBI Sequence Read Archive SRP103141.

NCBI BioProject accession number PRJNA381823.

## Supporting information


**Fig. S1** Predicted duplications and deletions in chromosomes 1‐14 for 18 strains of *D. septosporum*.

**Fig. S2** Initial evidence for a reciprocal chromosome translocation in the NZE10 genome. (A) Assembled contigs from the SLV genome were aligned with NZE10 reference chromosomes (scaffolds). Two contigs (circled) mapped to both chromosomes 5 and 13 of the NZE10 reference genome. This was found in many of the other genome sequences. (B) Visualisation of reads from the ALP3 genome mapped onto a region of chromosome 13 show a gap, in which mate pairs are mapped to chromosome 5.

**Fig. S3** A reciprocal translocation involving chromosomes 5 and 13 in the NZE10 genome. (A) The reciprocal translation was centred on an identical sequence (GCGCGGT) found at positions 1459800‐1459806 in NZE10 chromosome 5 and 717926‐717932 in chromosome 13. Chromosomes 5 and 13 are shaded grey and pale blue respectively with ends coloured to distinguish the two arms in each case. Coloured sequences surrounding the breakpoint indicate which arm they are from. (B) In strains from regions other than Australasia, the two long sections of NZE10 chromosomes 5 and 13 are joined to make a 2.2 Mb chromosome and two short sections to make a 1.4 Mb chromosome. Sequences around the common 7 bp sequence are shown for strain ALP3 as an example. (C, D) Pairs of divergently transcribed genes straddle the breakpoints on NZE10 chromosomes 5 (C) and 13 (D). A GC content of about 70% was seen at the breakpoint regions (50 bp sliding window) as shown by the %GC (blue) profiles.

**Fig. S4** Alignment of pathway regulator AflR from 19 *D.* septosporum strains. Amino acid changes compared to strain NZE10 are highlighted in blue (these sites are also variant between AflR sequences of *D. septosporum*, *Cladosporium fulvum*, *Aspergillus parasiticus* and *Aspergillus nidulans*; (Chettri *et al*., 2013)) or in green (at sites conserved between those four species). The Zn_2_Cys_6_ zinc binuclear domain is highlighted in pink; the linker sequence thought to determine DNA binding specificity in grey; the acidic glutamine rich motif in yellow and C terminal arginine residues implicated in AflJ binding in red.

**Fig. S5** Secondary structure predictions for AflR from *D. septosporum* NZE10 and ALP3. Pairwise alignment predicted by HHpred. The arrow indicates the location of the N349K polymorphism in ALP3.

**Table S1** Transposable elements in the *Dothistroma septosporum* genomes.

**Table S2** Genes deleted in the 18 genomes compared to *Dothistroma septosporum* NZE10.

**Table S3** Genes deleted from chromosome 14 and their expression levels in NZE10.

**Table S4** Single Nucleotide Polymorphisms (SNPs) in dothistromin genes, grouped by dothistromin gene loci.

**Table S5** Deleted genes on *Dothistroma septosporum* chromosome 12.

**Table S6** Gene duplications predicted by CNV (copy number variant) analysis.

**Table S7** (a) Polymerase Chain Reaction (PCR) primers used for verification of 5:13 translocation (b) Primers used for copy number variant (CNV) verification (quantitative PCR [qPCR]).
