# Supplementary material for: Global population genomics of the forest pathogen Dothistroma septosporum reveal chromosome duplications in high dothistromin‐producing strains
Source: Mol Plant Pathol. 2019 Apr 1;20(6):784–99. doi: 10.1111/mpp.12791 (PMC6637865; doi:10.1111/mpp.12791)
Supplement: Supplementary file 1 — Fig. S1 Predicted duplications and deletions in chromosomes 1‐14 for 18 strains of D. septosporum. [file MPP-20-784-s001.pdf]

Fig. S1 Predicted duplications and deletions in chromosomes 1-14 for 18 strains of *D. septosporum*. Predictions are from sequence read copy number variations.

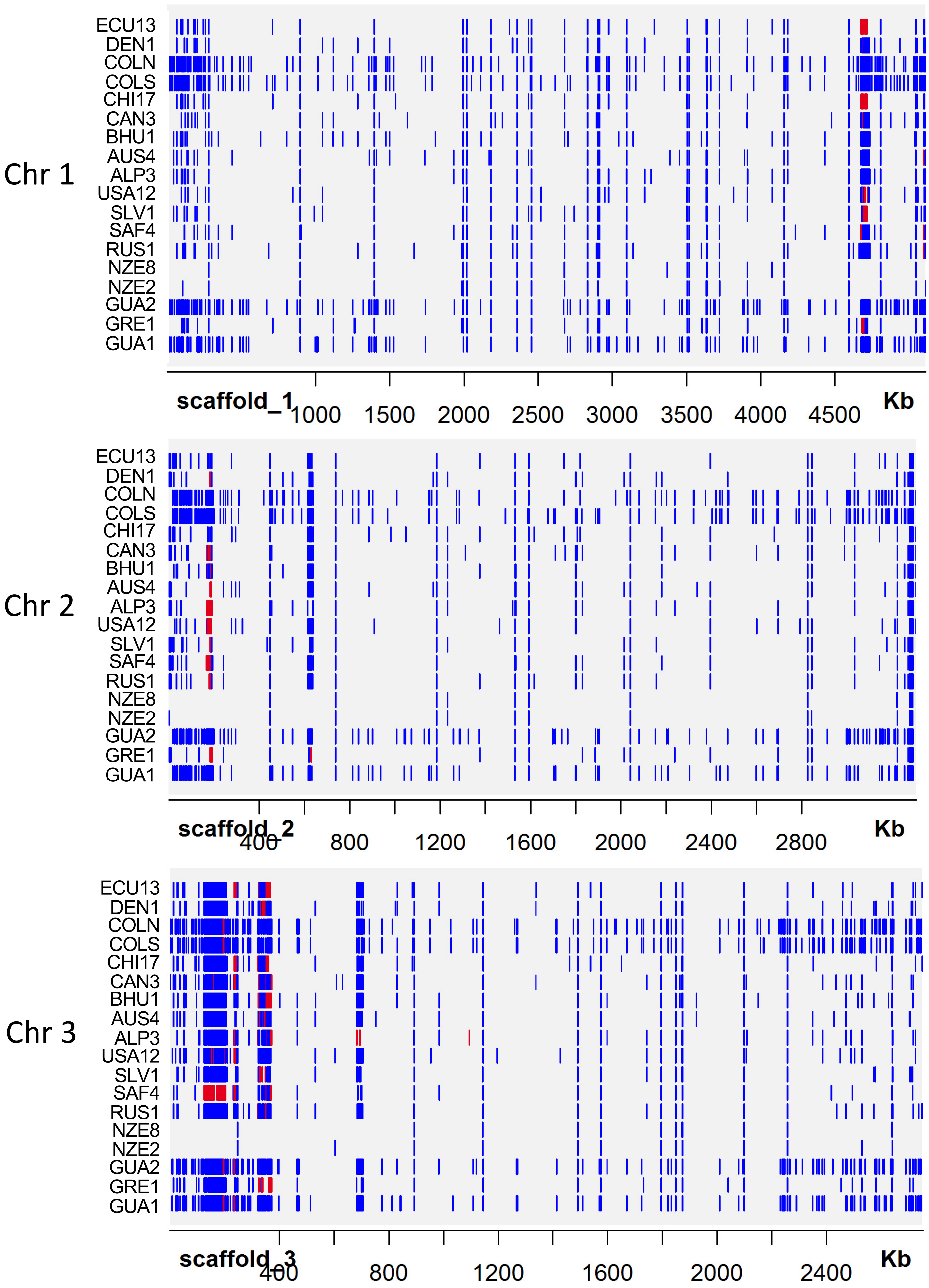

Red: duplicated; blue: deleted

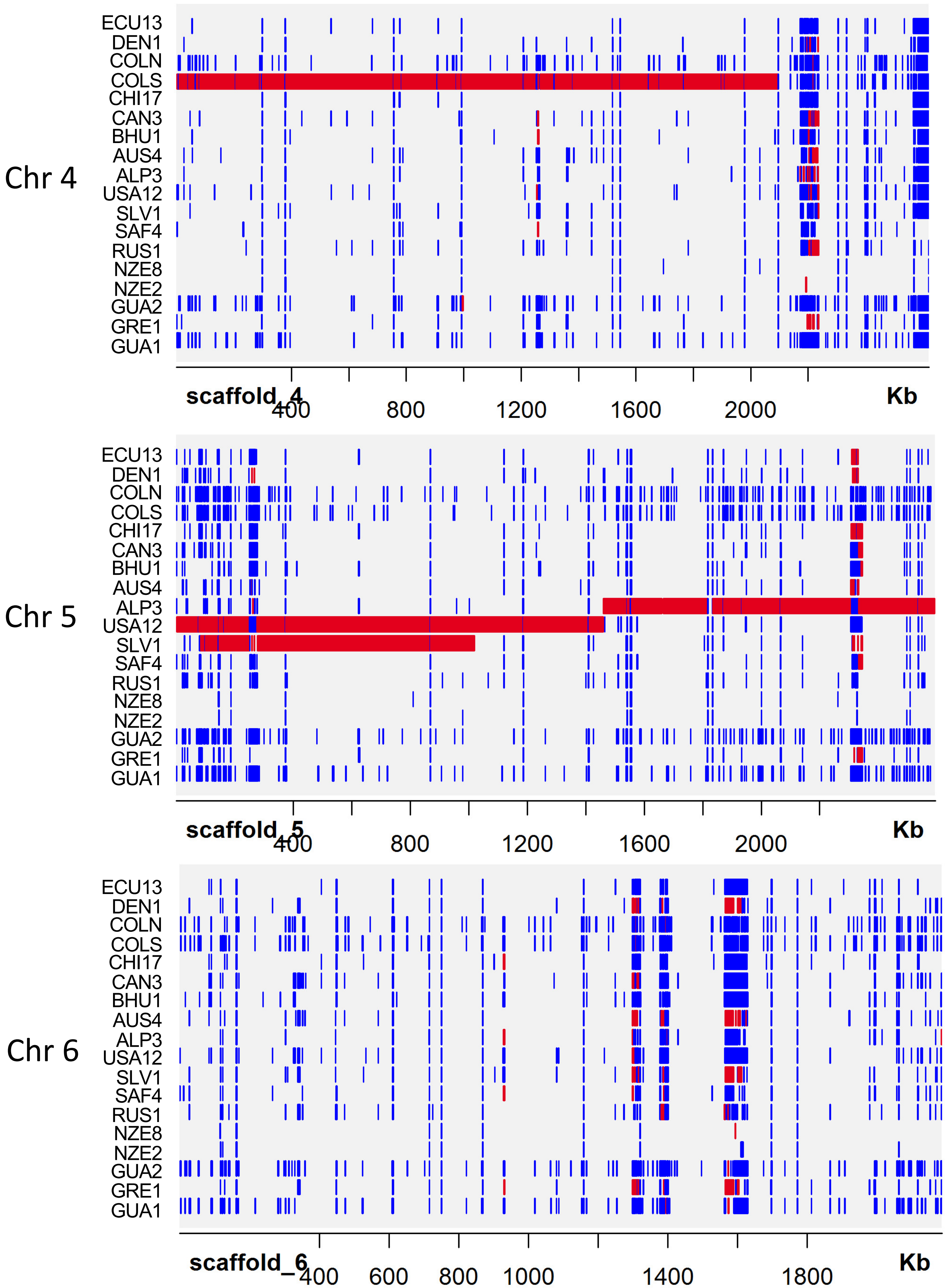

Red: duplicated; blue: deleted

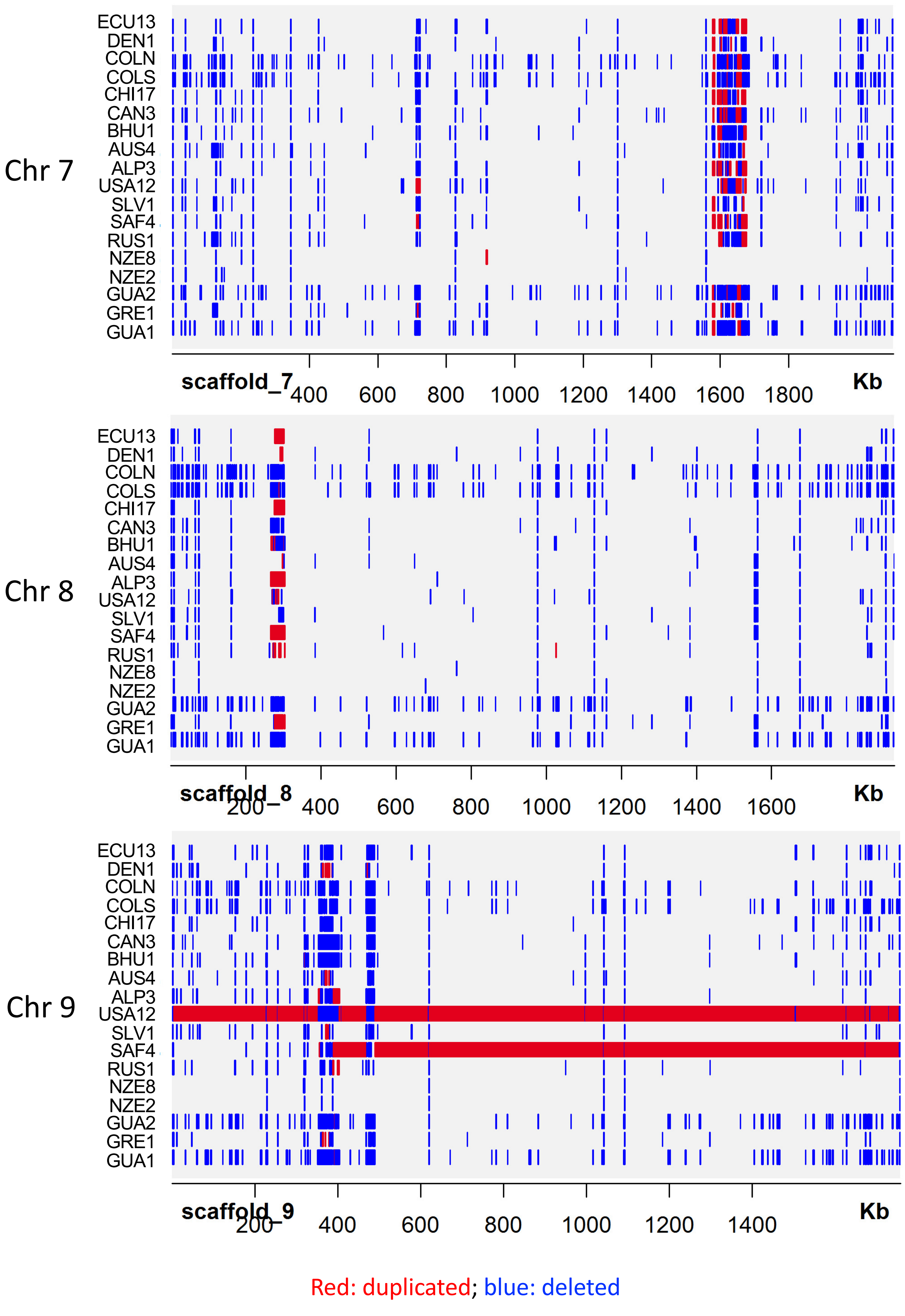
Chr 7
ECU13
DEN1
COLN
COLS
CHI17
CAN3
BHU1
AUS4
ALP3
USA12
SLV1
SAF4
RUS1
NZE8
NZE2
GUA2
GRE1
GUA1
scaffold_7
400
600
800
1000
1200
1400
1600
1800
Kb
Chr 8
ECU13
DEN1
COLN
COLS
CHI17
CAN3
BHU1
AUS4
ALP3
USA12
SLV1
SAF4
RUS1
NZE8
NZE2
GUA2
GRE1
GUA1
scaffold_8
200
400
600
800
1000
1200
1400
1600
Kb
Chr 9
ECU13
DEN1
COLN
COLS
CHI17
CAN3
BHU1
AUS4
ALP3
USA12
SLV1
SAF4
RUS1
NZE8
NZE2
GUA2
GRE1
GUA1
scaffold_9
200
400
600
800
1000
1200
1400
Kb
Red: duplicated; blue: deleted

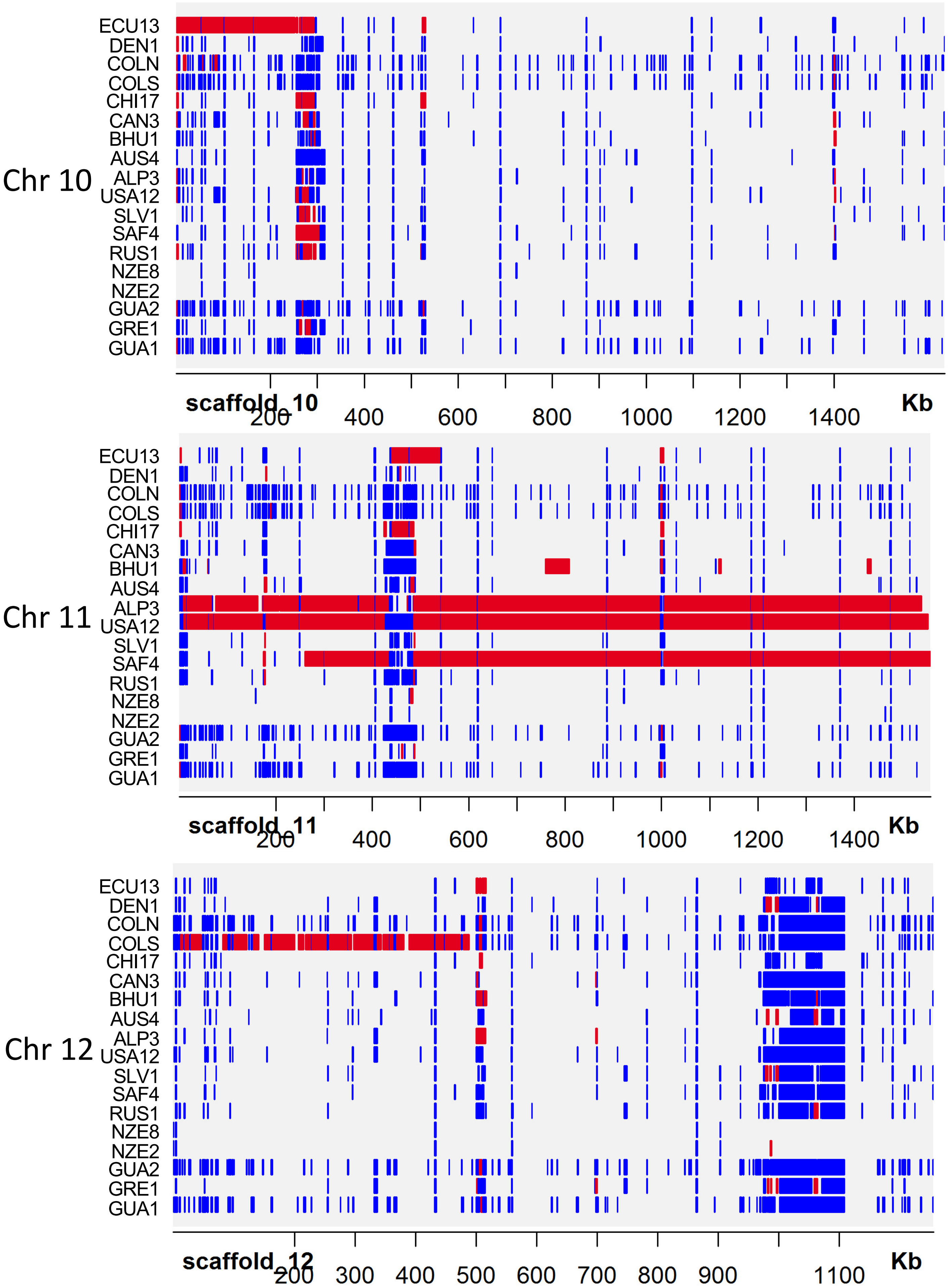

Red: duplicated; blue: deleted

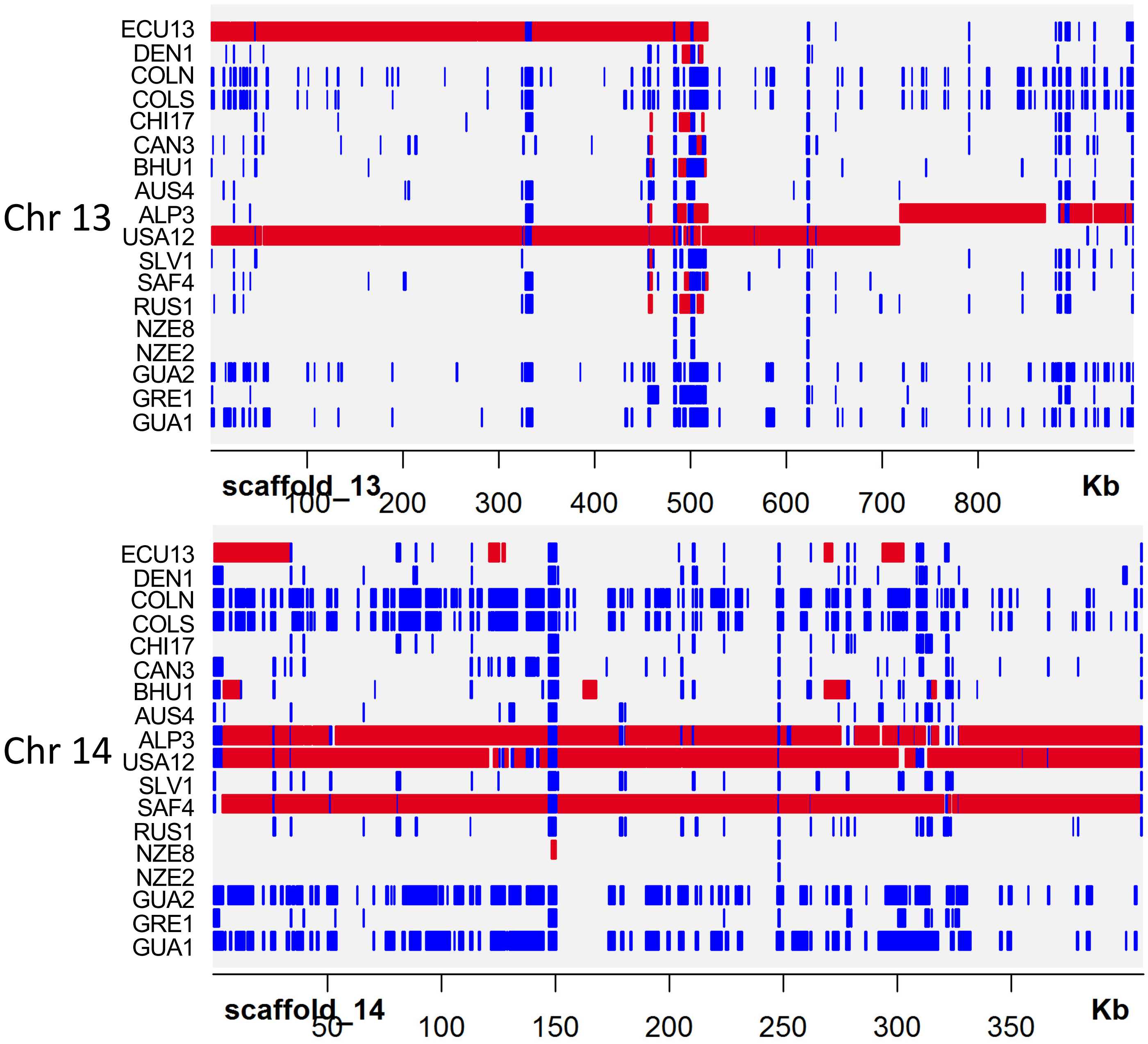

Red: duplicated; blue: deleted
