# Supplementary material for: Global population genomics of the forest pathogen Dothistroma septosporum reveal chromosome duplications in high dothistromin‐producing strains
Source: Mol Plant Pathol. 2019 Apr 1;20(6):784–99. doi: 10.1111/mpp.12791 (PMC6637865; doi:10.1111/mpp.12791)
Supplement: Supplementary file 2 — Fig. S2 Initial evidence for a reciprocal chromosome translocation in the NZE10 genome. (A) Assembled contigs from the SLV genome were aligned with NZE10 reference chromosomes (scaffolds). Two contigs (circled) mapped to both chromosomes 5 and 13 of the NZE10 reference genome. This was found in many of the other genome sequences. (B) Visualisation of reads from the ALP3 genome mapped onto a region of chromosome 13 show a gap, in which mate pairs are mapped to chromosome 5. [file MPP-20-784-s002.pdf]

Fig. S2

Initial evidence for a 5:13 reciprocal chromosome translocation in the NZE10 genome.

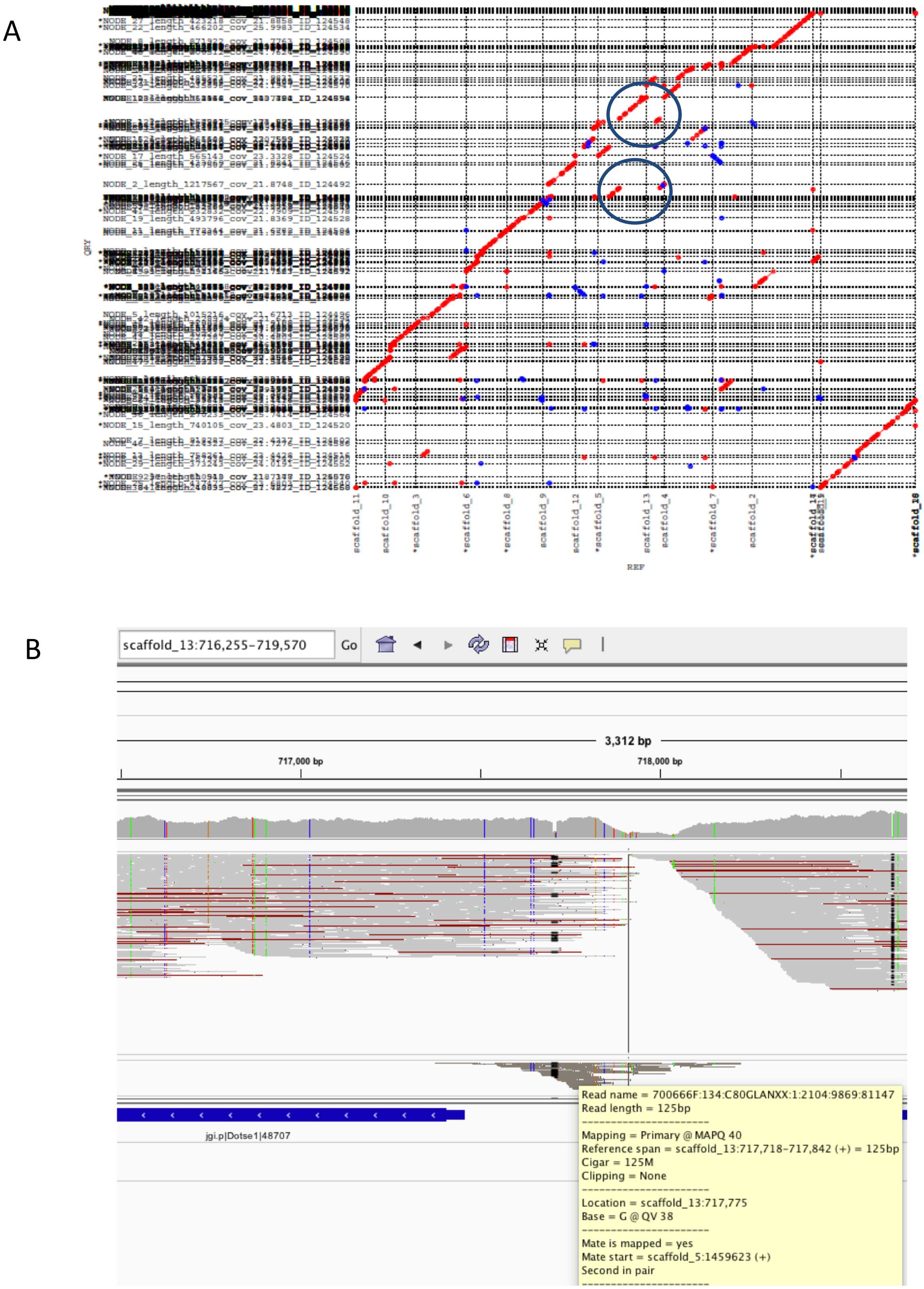

A) Assembled contigs from the SLV genome were aligned with the reference chromosomes (scaffolds). Two of the contigs (circled) mapped to both chromosomes 5 and 13 of the NZE10 reference genome. This was found in many of the other genome sequences.

B) Visualisation of reads from the ALP3 genome mapped onto a region of chromosome 13 show a gap, in which mate-pairs are mapped to chromosome 5.
