# Supplementary material for: Global population genomics of the forest pathogen Dothistroma septosporum reveal chromosome duplications in high dothistromin‐producing strains
Source: Mol Plant Pathol. 2019 Apr 1;20(6):784–99. doi: 10.1111/mpp.12791 (PMC6637865; doi:10.1111/mpp.12791)
Supplement: Supplementary file 3 — Fig. S3 A reciprocal translocation involving chromosomes 5 and 13 in the NZE10 genome. (A) The reciprocal translation was centred on an identical sequence (GCGCGGT) found at positions 1459800‐1459806 in NZE10 chromosome 5 and 717926‐717932 in chromosome 13. Chromosomes 5 and 13 are shaded grey and pale blue respectively with ends coloured to distinguish the two arms in each case. Coloured sequences surrounding the breakpoint indicate which arm they are from. (B) In strains from regions other than Australasia, the two long sections of NZE10 chromosomes 5 and 13 are joined to make a 2.2 Mb chromosome and two short sections to make a 1.4 Mb chromosome. Sequences around the common 7 bp sequence are shown for strain ALP3 as an example. (C, D) Pairs of divergently transcribed genes straddle the breakpoints on NZE10 chromosomes 5 (C) and 13 (D). A GC content of about 70% was seen at the breakpoint regions (50 bp sliding window) as shown by the %GC (blue) profiles. [file MPP-20-784-s003.pdf]

Fig. S3 A,B

A reciprocal translocation involving chromosomes 5 and 13 in the NZE10 genome.

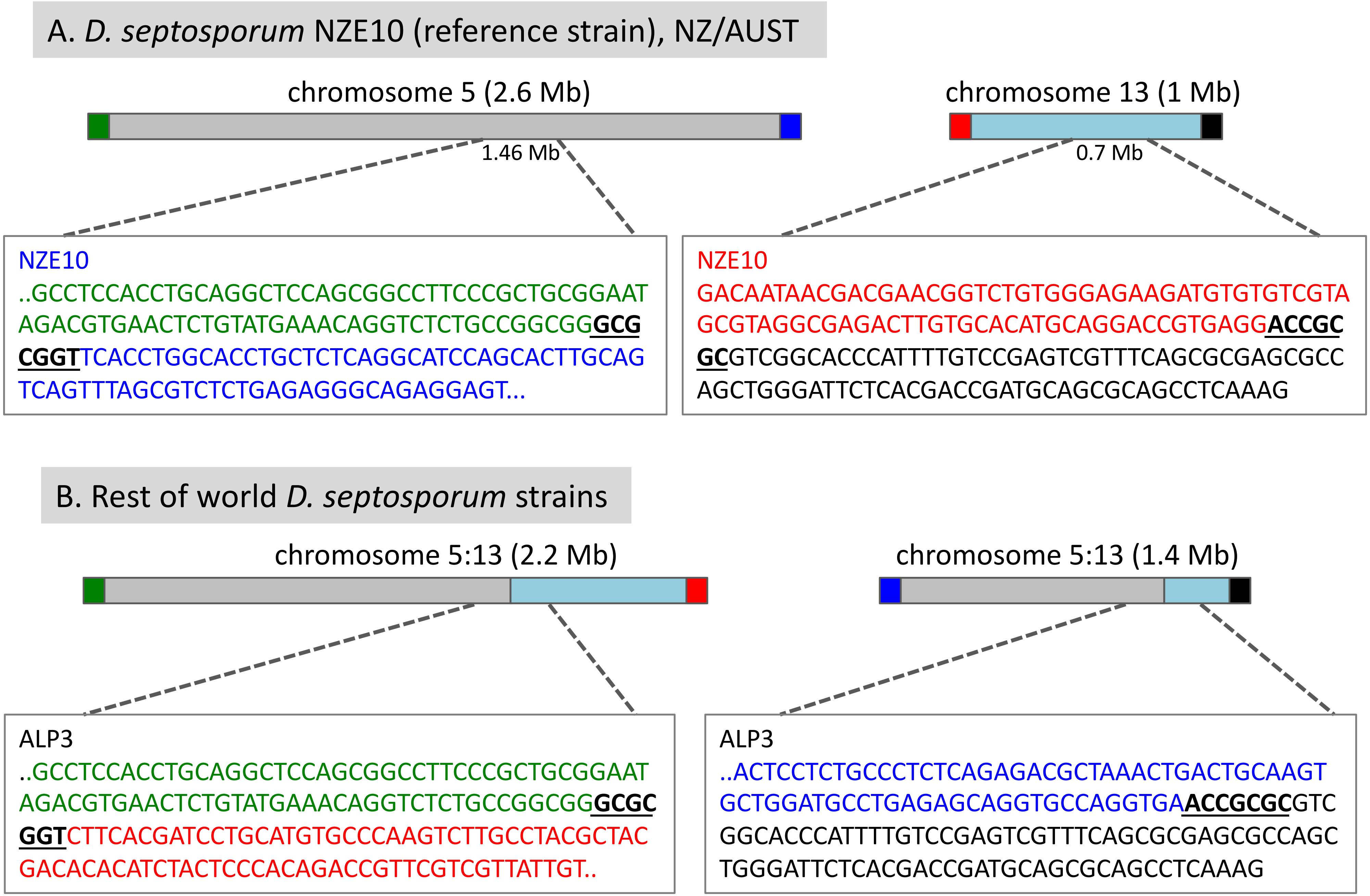

Fig. S3 C,D

A reciprocal translocation involving chromosomes 5 and 13 in the NZE10 genome.

Expression data are from Bradshaw et al. (2016)

C. NZE10 chromosome 5

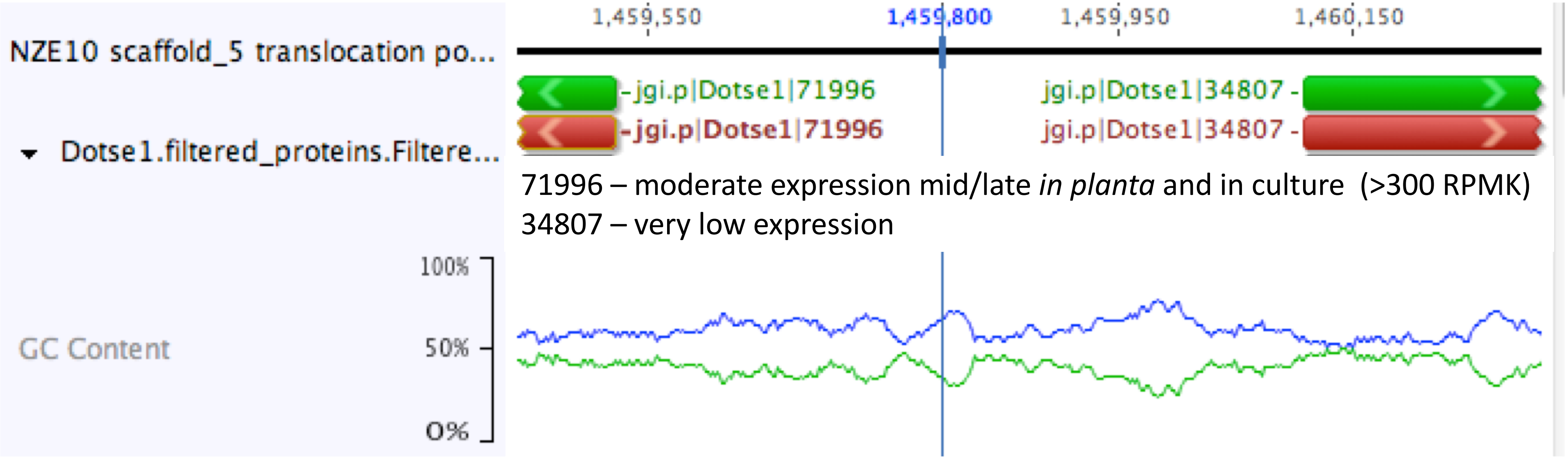

D. NZE10 chromosome 13

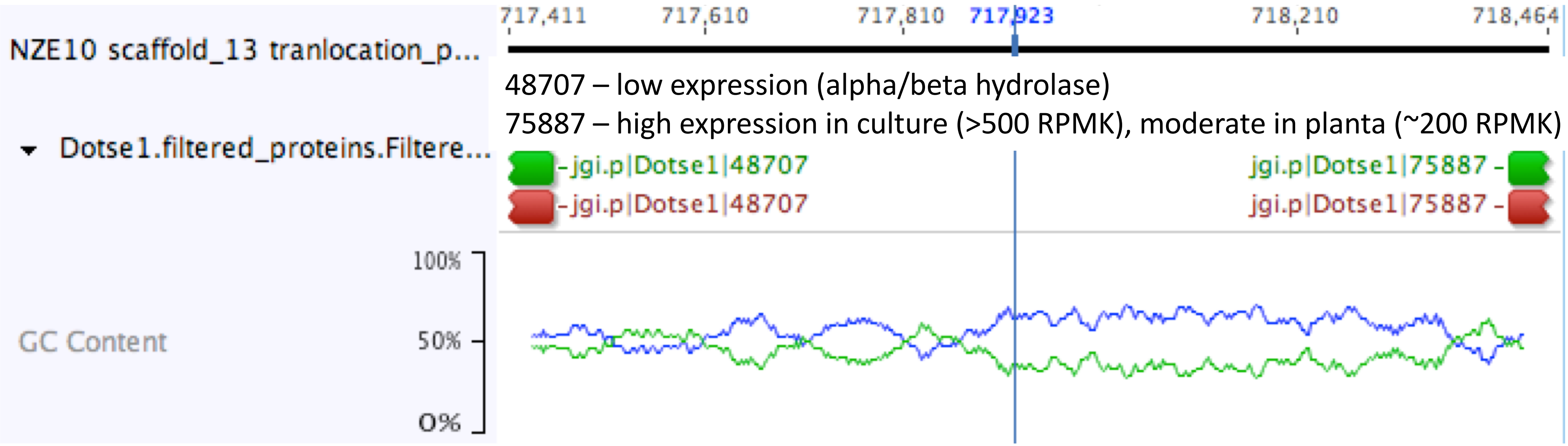

Fig S3 legend.

A reciprocal translocation involving chromosomes 5 and 13 in the NZE10 genome.

A) The reciprocal translation was centred on an identical sequence (GCGCGGT) found at positions 1459800-1459806 in NZE10 chromosome 5 and 717926-717932 in chromosome 13. Chromosomes 5 and 13 are shaded grey and pale blue respectively with ends coloured to distinguish the two arms in each case. Coloured sequences surrounding the breakpoint indicate which arm they are from.

B) In strains from regions other than Australasia, the two long sections of NZE10 chromosomes 5 and 13 are joined to make a 2.2 Mb chromosome and two short sections to make a 1.4 Mb chromosome. Sequences around the common 7 bp sequence are shown for strain ALP3 as an example.

C,D) Pairs of divergently transcribed genes straddle the breakpoints on NZE10 chromosomes 5 (C) and 13 (D). A GC content of about 70% was seen at the breakpoint regions (50 bp sliding window) as shown by the %GC (blue) profiles.
