# Supplementary material for: Global population genomics of the forest pathogen Dothistroma septosporum reveal chromosome duplications in high dothistromin‐producing strains
Source: Mol Plant Pathol. 2019 Apr 1;20(6):784–99. doi: 10.1111/mpp.12791 (PMC6637865; doi:10.1111/mpp.12791)
Supplement: Supplementary file 4 — Fig. S4 Alignment of pathway regulator AflR from 19 D. septosporum strains. Amino acid changes compared to strain NZE10 are highlighted in blue (these sites are also variant between AflR sequences of D. septosporum, Cladosporium fulvum, Aspergillus parasiticus and Aspergillus nidulans; (Chettri et al., 2013)) or in green (at sites conserved between those four species). The Zn2Cys6 zinc binuclear domain is highlighted in pink; the linker sequence thought to determine DNA binding specificity in grey; the acidic glutamine rich motif in yellow and C terminal arginine residues implicated in AflJ binding in red. [file MPP-20-784-s004.pdf]

Figure S4. Alignment of pathway regulator DsAflR from 19 *D. septosporum* strains

```
0_NZE10      1 MPESRGSESSGSTSRRASGTQHIATPKLKDSCTACATSKVKCSKDKPTCARCTRRGLTCDYGLSKRTGRTSHATAQKQQQQRQQQQQSQQEAADRRVSQS 100
1_ALP3       1 MPESRGSESSGSTSRRASGTQHIATPKLKDSCTACATSKVKCSKDKPTCARCTRRGLTCDYGLSKRTGRTSHATAQKQQQQRQQQQQSQQEAADRRVSQS 100
2_AUS4       1 MPESRGSESSGSTSRRASGTQHIATPKLKDSCTACATSKVKCSKDKPTCARCTRRGLTCDYGLSKRTGRTSHATAQKQQQQRQQQQQSQQEAADRRVSQS 100
3_BHU1       1 MPESRGSESSGSTSRRASGTQHIATPKLKDSCTACATSKVKCSKDKPTCARCTRRGLTCDYGLSKRTGRTSHATAQKQQQQRQQQQQSQQEAADRRVSQS 100
4_CAN3       1 MPESRGSESSGSTSRRASGTQHIATPKLKDSCTACATSKVKCSKDKPTCARCTRRGLTCDYGLSKRTGRTSHATAQKQQQQRQQQQQSQQEAADRRVSQS 100
5_CHI17      1 MPESRGSESSGSTSRRASGTQHIATPKLKDSCTACATSKVKCSKDKPTCARCTRRGLTCDYGLSKRTGRTSHATAQKQQQQRQQQQQSQQEAADRRVSQS 100
6_COLN       1 MPESRGSESSGSTSRRASGTQHIATPKLKDSCTACATSKVKCSKDKPTCARCTRRGLTCDYGLSKRTGRTSHATAQKQQQQRQQQQQSQQEAADRRVSQS 100
7_COLS       1 MPESRGSESSGSTSRRASGTQHIATPKLKDSCTACATSKVKCSKDKPTCARCTRRGLTCDYGLSKRTGRTSHATAQKQQQQRQQQQQSQQEAADRRVSQS 100
8_DEN1       1 MPESRGSESSGSTSRRASGTQHIATPKLKDSCTACATSKVKCSKDKPTCARCTRRGLTCDYGLSKRTGRTSHATAQKQQQQRQQQQQSQQEAADRRVSQS 100
9_ECU13      1 MPESRGSESSGSTSRRASGTQHIATPKLKDSCTACATSKVKCSKDKPTCARCTRRGLTCDYGLSKRTGRTSHATAQKQQQQRQQQQQSQQEAADRRVSQS 100
10_GUA1      1 MPESRGSESSGSTSRRASGTQHIATPKLKDSCTACATSKVKCSKDKPTCARCTRRGLTCDYGLSKRTGRTSHATAQKQQQQRQQQQQSQQEAADRRVSQS 100
11_GRE1      1 MPESRGSESSGSTSRRASGTQHIATPKLKDSCTACATSKVKCSKDKPTCARCTRRGLTCDYGLSKRTGRTSHATAQKQQQQRQQQQQSQQEAADRRVSQS 100
12_GUA2      1 MPESRGSESSGSTSRRASGTQHIATPKLKDSCTACATSKVKCSKDKPTCARCTRRGLTCDYGLSKRTGRTSHATAQKQQQQRQQQQQSQQEAADRRVSQS 100
13_NZE2      1 MPESRGSESSGSTSRRASGTQHIATPKLKDSCTACATSKVKCSKDKPTCARCTRRGLTCDYGLSKRTGRTSHATAQKQQQQRQQQQQSQQEAADRRVSQS 100
14_NZE8      1 MPESRGSESSGSTSRRASGTQHIATPKLKDSCTACATSKVKCSKDKPTCARCTRRGLTCDYGLSKRTGRTSHATAQKQQQQRQQQQQSQQEAADRRVSQS 100
15_RUS1      1 MPESRGSESSGSTSRRASGTQHIATPKLKDSCTACATSKVKCSKDKPTCARCTRRGLTCDYGLSKRTGRTSHATAQKQQQQRQQQQQSQQEAADRRVSQS 100
16_SAF4      1 MPESRGSESSGSTSRRASGTQHIATPKLKDSCTACATSKVKCSKDKPTCARCTRRGLTCDYGLSKRTGRTSHATAQKQQQQRQQQQQSQQEAADRRVSQS 100
17_SLV1      1 MPESRGSESSGSTSRRASGTQHIATPKLKDSCTACATSKVKCSKDKPTCARCTRRGLTCDYGLSKRTGRTSHATAQKQQQQRQQQQQSQQEAADRRVSQS 100
18_USA12     1 MPESRGSESSGSTSRRASGTQHIATPKLKDSCTACATSKVKCSKDKPTCARCTRRGLTCDYGLSKRTGRTSHATAQKQQQQRQQQQQSQQEAADRRVSQS 100
               ****************************************************************************************************

0_NZE10    101 AVPVANMLPDVDEFISPMSLQESSILSPTLMPDLSTSGSQAGLDQWNSDLWSTMFAPNEPSSNNTPANTMQTHGGNGNDIDHLFNDLTSYSMLGEPTDTA 200
1_ALP3     101 AVPVANMLPDVDEFISPMSLQESSILSPTLMPDLSTSGSQAGLDQWNSDLWSTMFAPNEPSSNNTPANTMQTHGGNGNDIDHLFNDLTSYSMLGEPTDTA 200
2_AUS4     101 AVPVANMLPDVDEFISPMSLQESSILSPTLMPDLSTSGSQAGLDQWNSDLWSTMFAPNEPSSNNTPANTMQTHGGNGNDIDHLFNDLTSYSMLGEPTDTA 200
3_BHU1     101 AVPVANMLPDVDEFISPMSLQESSILSPALMPDLSTSGSQAGLDQWNSDLWSTMFAPNEPSSNNTPANTMQTHGGNGNDIDHLFNDLTSYSMLGEPTDTA 200
4_CAN3     101 AVPVANMLPDVDEFISPMSLQESSILSPTLMPDLSTSGSQAGLDQWNSDLWSTMFAPNEPSSNNTPANTMQTHGGNGNDIDHLFNDLTSYSMLGEPTDTA 200
5_CHI17    101 AVPVANMLPDVDEFISPMSLQESSILSPTLMPDLSTSGSQAGLDQWNSDLWSTMFAPNEPSSNNTPANTMQTHGGNGNDIDHLFNDLTSYSMLGEPTDTA 200
6_COLN     101 AVPVANMLPDVDEFISPMSLQESSILSPTLMPDLSTSGSQAGLDQWNSDLWSTMFAPNEPSSNNTPANTMQTHGGNGNDIDHLFNDLTSYSMLGEPTDTA 200
7_COLS     101 AVPVANMLPDVDEFISPMSLQESSILSPTLMPDLSTSGSQAGLDQWNSDLWSTMFAPNEPSSNNTPANTMQTHGGNGNDIDHLFNDLTSYSMLGEPTDTA 200
8_DEN1     101 AVPVANMLPDVDEFISPMSLQESSILSPTLMPDLSTSGSQAGLDQWNSDLWSTMFAPNEPSSNNTPANTMQTHGGNGNDIDHLFNDLTSYSMLGEPTDTA 200
9_ECU13    101 AVPVANMLPDVDEFISPMSLQESSILSPTLMPDLSTSGSQAGLDQWNSDLWSTMFAPNEPSSNNTPANTMQTHGGNGNDIDHLFNDLTSYSMLGEPTDTA 200
10_GUA1    101 AVPVANMLPDVDEFISPMSLQESSILSPTLMPDLSTSGSQAGLDQWNSDLWSTMFTPNEPSSNNTPANTMQTHGGNGNDIDHLFNDLTSYSMLGEPTDTA 200
11_GRE1    101 AVPVANMLPDVDEFISPMSLQESSILSPTLMPDLSTSGSQAGLDQWNSDLWSTMFAPNEPSSNNTPANTMQTHGGNGNDIDHLFNDLTSYSMLGEPTDTA 200
12_GUA2    101 AVPVANMLPDVDEFISPMSLQESSILSPTLMPDLSTSGSQAGLDQWNSDLWSTMFAPNEPSSNNTPANTMQTHGGNGNDIDHLFNDLTSYSMLGEPTDTA 200
13_NZE2    101 AVPVANMLPDVDEFISPMSLQESSILSPTLMPDLSTSGSQAGLDQWNSDLWSTMFAPNEPSSNNTPANTMQTHGGNGNDIDHLFNDLTSYSMLGEPTDTA 200
14_NZE8    101 AVPVANMLPDVDEFISPMSLQESSILSPTLMPDLSTSGSQAGLDQWNSDLWSTMFAPNEPSSNNTPANTMQTHGGNGNDIDHLFNDLTSYSMLGEPTDTA 200
15_RUS1    101 AVPVANMLPDVDEFISPMSLQESSILSPTLMPDLSTSGSQAGLDQWNSDLWSTMFAPNEPSSNNTPANTMQTHGGNGNDIDHLFNDLTSYSMLGEPTDTA 200
16_SAF4    101 AVPVANMLPDVDEFISPMSLQESSILSPTLMPDLSTSGSQAGLDQWNSDLWSTMFAPNEPSSNNTPANTMQTHGGNGNDIDHLFNDLTSYSMLGEPTDTA 200
17_SLV1    101 AVPVANMLPDVDEFISPMSLQESSILSPTLMPDLSTSGSQAGLDQWNSDLWSTMFAPNEPSSNNTPANTMQTHGGNGNDIDHLFNDLTSYSMLGEPTDTA 200
18_USA12   101 AVPVANMLPDVDEFISPMSLQESSILSPTLMPDLSTSGSQAGLDQWNSDLWSTMFAPNEPSSNNTPANTMQTHGGNGNDIDHLFNDLTSYSMLGEPTDTA 200
               ****************************v**************************v********************************************
```

```
0_NZE10   201 MSNQHTHTFSDTSSSISPFDQLTSVSDMGSQDFSNIDLSMTNCTPAIQQSVEPANCCLTVALGFMTQLCATASSSCTMPGSHNGNTTLPTIDSVITENRQ 300
1_ALP3    201 MSNQHTHTFSDTSSSISPFDQLTSVSDMGSQDFSNIDLSMANCTPAIQQSVEPANCCLTVALGFMTQLCATASSSCTMPGSHNGNTTLPTIDSVITENRQ 300
2_AUS4    201 MSNQHTHTFSDTSSSISPFDQLTSVSDMGSQDFSNIDLSMANCTPAIQQSVEPANCCLTVALGFMTQLCATASSSCTMPGSHNGNTTLPTIDSVITENRQ 300
3_BHU1    201 MSNQHTHTFSDTSSSISPFDQLTSVSDMGSQDFSNIDLSMANCTPAIQQSVEPANCCLTVALGFMTQLCATASSSCTMPGSHNGNTTLPTIDSVITENRQ 300
4_CAN3    201 MSNQHTHTFSDTSSSISPFDQLTSVSDMGSQDFSNIDLSMANCTPAIQQSVEPANCCLTVALGFMTQLCATASSSCTMPGSHNGNTTLPTIDSVITENRQ 300
5_CHI17   201 MSNQHTHTFSDTSSSISPFDQLTSVSDMGSQDFSNIDLSMANCTPAIQQSVEPANCCLTVALGFMTQLCATASSSCTMPGSHNGNTTLPTIDSVITENRQ 300
6_COLN    201 MSNQHTHTFSDTSSSTSPFDQLTSVSDMGSQDFSNIDLSMANCTPAIQQSVEPANCCLTVALGFMTQLCATASSSCTMPGSHNGNTTLPTIDSVITENRQ 300
7_COLS    201 MSNQHTHTFSDTSSSTSPFDQLTSVSDMGSQDFSNIDLSMANCTPAIQQSVEPANCCLTVALGFMTQLCATASSSCTMPGSHNGNTTLPTIDSVITENRQ 300
8_DEN1    201 MSNQHTHTFSDTSSSISPFDQLTSVSDMGSQDFSNIDLSMANCTPAIQQSVEPANCCLTVALGFMTQLCATASSSCTMPGSHNGNTTLPTIDSVITENRQ 300
9_ECU13   201 MSNQHTHTFSDTSSSISPFDQLTSVSDMGSQDFSNIDLSMANCTPAIQQSVEPANCCLTVALGFMTQLCATASSSCTMPGSHNGNTTLPTIDSVITENRQ 300
10_GUA1   201 MSNQHTHTFSDTSSSISPFDQLTSVSDMGSQDFSNIDLSMANCTPAIQQSVEPANCCLTVALGFMTQLCATASSSCTMPGSHNGNTTLPTIDSVITENRQ 300
11_GRE1   201 MSNQHTHTFSDTSSSISPFDQLTSVSDMGSQDFSNIDLSMANCTPAIQQSVEPANCCLTVALGFMTQLCATASSSCTMPGSHNGNTTLPTIDSVITENRQ 300
12_GUA2   201 MSNQHTHTFSDTSSSTSPFDQLTSVSDMGSQDFSNIDLSMANCTPAIQQSVEPANCCLTVALGFMTQLCATASSSCTMPGSHNGNTTLPTIDSVITENRQ 300
13_NZE2   201 MSNQHTHTFSDTSSSISPFDQLTSVSDMGSQDFSNIDLSMTNCTPAIQQSVEPANCCLTVALGFMTQLCATASSSCTMPGSHNGNTTLPTIDSVITENRQ 300
14_NZE8   201 MSNQHTHTFSDTSSSISPFDQLTSVSDMGSQDFSNIDLSMTNCTPAIQQSVEPANCCLTVALGFMTQLCATASSSCTMPGSHNGNTTLPTIDSVITENRQ 300
15_RUS1   201 MSNQHTHTFSDTSSSISPFDQLTSVSDMGSQDFSNIDLSMANCTPAIQQSVEPANCCLTVALGFMTQLCATASSSCTMPGSHNGNTTLPTIDSVITENRQ 300
16_SAF4   201 MSNQHTHTFSDTSSSISPFDQLTSVSDMGSQDFSNIDLSMANCTPAIQQSVEPANCCLTVALGFMTQLCATASSSCTMPGSHNGNTTLPTIDSVITENRQ 300
17_SLV1   201 MSNQHTHTFSDTSSSISPFDQLTSVSDMGSQDFSNIDLSMANCTPAIQQSVEPANCCLTVALGFMTQLCATASSSCTMPGSHNGNTTLPTIDSVITENRQ 300
18_USA12  201 MSNQHTHTFSDTSSSISPFDQLTSVSDMGSQDFSNIDLSMANCTPAIQQSVEPANCCLTVALGFMTQLCATASSSCTMPGSHNGNTTLPTIDSVITENRQ 300
              ***************v************************v***********************************************************

0_NZE10   301 IVDQIVKILECPCSHDEYLLTIVHLVVFKVMAWYAAAAREKPSLAEEINWTDQQSGRPRSRTHSEEVLRFPPSIDGYSSDGSDNGRMAAQLVLSELHRVQ 400
1_ALP3    301 IVDQIVKILECPCSHDEYLLTIVHLVVFKVMAWYAAAAREKPSLAEEIKWTDQQSGRPRSRTHSEEVLRFPPSIDGYSSDGSDNGRMAAQLVLSELHRVQ 400
2_AUS4    301 IVDQIVKILECPCSHDEYLLTIVHLVVFKVMAWYAAAAREKPSLAEEINWTDQQSGRPRSRTHSEEVLRFPPSIDGYSSDGSDNGRMAAQLVLSELHRVQ 400
3_BHU1    301 IVDQIVKILECPCSHDEYLLTIVHLVVFKVMAWYAAAAREKPSLAEEINWTDQQSGRPRSRTHSEEVLRFPPSIDGYSSDGSDNGRMAAQLVLSELHRVQ 400
4_CAN3    301 IVDQIVKILECPCSHDEYLLTIVHLVVFKVMAWYAAAAREKPSLAEEINWTDQQSGRPRSRTHSEEVLRFPPSIDGYSSDGSDNGRMAAQLVLSELHRVQ 400
5_CHI17   301 IVDQIVKILECPCSHDEYLLTIVHLVVFKVMAWYAAAAREKPSLAEEINWTDQQSGRPRSRTHSEEVLRFPPSIDGYSSDGSDNGRMAAQLVLSELHRVQ 400
6_COLN    301 IVDQIVKILECPCSHDEYLLTIVHLVVFKVMAWYAAAAREKPSLAEEINWTDQQSDRPRSRTHSEEVLRFPPSIDGYSSDGSDNGRMAAQLVLSELHRVQ 400
7_COLS    301 IVDQIVKILECPCSHDEYLLTIVHLVVFKVMAWYAAAAREKPSLAEEINWTDQQSDRPRSRTHSEEVLRFPPSIDGYSSDGSDNGRMAAQLVLSELHRVQ 400
8_DEN1    301 IVDQIVKILECPCSHDEYLLTIVHLVVFKVMAWYAAAAREKPSLAEEINWTDQQSGRPRSRTHSEEVLRFPPSIDGYSSDGSDNGRMAAQLVLSELHRVQ 400
9_ECU13   301 IVDQIVKILECPCSHDEYLLTIVHLVVFKVMAWYAAAAREKPSLAEEINWTDQQSGRPRSRTHSEEVLRFPPSIDGYSSDGSDNGRMAAQLVLSELHRVQ 400
10_GUA1   301 IVDQIVKILECPCSHDEYLLTIVHLVVFKVMAWYAAAAREKPSLAEEINWTDQQSDRPRSRTHSEEVLRFPPSIDGYSSDGSDNGRMAAQLVLSELHRVQ 400
11_GRE1   301 IVDQIVKILECPCSHDEYLLTIVHLVVFKVMAWYAAAAREKPSLAEEINWTDQQSGRPRSRTHSEEVLRFPPSIDGYSSDGSDNGRMAAQLVLSELHRVQ 400
12_GUA2   301 IVDQIVKILECPCSHDEYLLTIVHLVVFKVMAWYAAAAREKPSLAEEINWTDQQSDRPRSRTHSEEVLRFPPSIDGYSSDGSDNGRMAAQLVLSELHRVQ 400
13_NZE2   301 IVDQIVKILECPCSHDEYLLTIVHLVVFKVMAWYAAAAREKPSLAEEINWTDQQSGRPRSRTHSEEVLRFPPSIDGYSSDGSDNGRMAAQLVLSELHRVQ 400
14_NZE8   301 IVDQIVKILECPCSHDEYLLTIVHLVVFKVMAWYAAAAREKPSLAEEINWTDQQSGRPRSRTHSEEVLRFPPSIDGYSSDGSDNGRMAAQLVLSELHRVQ 400
15_RUS1   301 IVDQIVKILECPCSHDEYLLTIVHLVVFKVMAWYAAAAREKPSLAEEINWTDQQSGRPRSRTHSEEVLRFPPSIDGYSSDGSDNGRMAAQLVLSELHRVQ 400
16_SAF4   301 IVDQIVKILECPCSHDEYLLTIVHLVVFKVMAWYAAAAREKPSLAEEINWTDQQSGRPRSRTHSEEVLRFPPSIDGYSSDGSDNGRMAAQLVLSELHRVQ 400
17_SLV1   301 IVDQIVKILECPCSHDEYLLTIVHLVVFKVMAWYAAAAREKPSLAEEINWTDQQSGRPRSRTHSEEVLRFPPSIDGYSSDGSDNGRMAAQLVLSELHRVQ 400
18_USA12  301 IVDQIVKILECPCSHDEYLLTIVHLVVFKVMAWYAAAAREKPSLAEEINWTDQQSGRPRSRTHSEEVLRFPPSIDGYSSDGSDNGRMAAQLVLSELHRVQ 400
              ************************************************C******v********************************************
```

```
0_NZE10    401 RLVNLLSQRLEGVRLRNHVASSGSSSSLESIGEDSVVGVSLSATAGSPLSSPTFDQLEADLRKRLRAVSFETIDVLRRS 479
1_ALP3     401 RLVNLLSQRLEGVRLRNHVASSGSSSSLESIGEDSVVGVSLSATAGSPLSSPTFDQLEADLRKRLRAVSFETIDVLRRS 479
2_AUS4     401 RLVNLLSQRLEGVRLRNHVASSGSSSSLESIGEDSVVGVSLSATAGSPLSSPTFDQLEADLRKRLRAVSFETIDVLRRS 479
3_BHU1     401 RLVNLLSQRLEGVRLRNHVASSGSSSSLESIGEDSVVGVSLSATAGSPLSSPTFDQLEADLRKRLRAVSFETIDVLRRS 479
4_CAN3     401 RLVNLLSQRLEGVRLRNHVASSGSSSSLESIGEDSVVGVSLSATAGSPLSSPTFDQLEADLRKRLRAVSFETIDVLRRS 479
5_CHI17    401 RLVNLLSQRLEGVRLRNHVASSGSSSSLESIGEDSVVGVSLSATAGSPLSSPTFDQLEADLRKRLRAVSFETIDVLRRS 479
6_COLN     401 RLVNLLSQRLEGVRLRNHVASSGSSSSLESIGEDSVVGVSLSATAGSPLSSPTFDQLEADLRKRLRAVSFETIDVLRRS 479
7_COLS     401 RLVNLLSQRLEGVRLRNHVASSGSSSSLESIGEDSVVGVSLSATAGSPLSSPTFDQLEADLRKRLRAVSFETIDVLRRS 479
8_DEN1     401 RLVNLLSQRLEGVRLRNHVASSGSSSSLESIGEDSVVGVSLSATAGSPLSSPTFDQLEADLRKRLRAVSFETIDVLRRS 479
9_ECU13    401 RLVNLLSQRLEGVRLRNHVASSGSSSSLESIGEDSVVGVSLSATAGSPLSSPTFDQLEADLRKRLRAVSFETIDVLRRS 479
10_GUA1    401 RLVNLLSQRLEGVRLRNHVASSGSSSSLESIGEDSVVGVSLSATAGSPLSSPTFDQLEADLRKRLRAVSFETIDVLRRS 479
11_GRE1    401 RLVNLLSQRLEGVRLRNHVASSGSSSSLESIGEDSVVGVSLSATAGSPLSSPTFDQLEADLRKRLRAVSFETIDVLRRS 479
12_GUA2    401 RLVNLLSQRLEGVRLRNHVASSGSSSSLESIGEDSVVGVSLSATAGSPLSSPTFDQLEADLRKRLRAVSFETIDVLRRS 479
13_NZE2    401 RLVNLLSQRLEGVRLRNHVASSGSSSSLESIGEDSVVGVSLSATAGSPLSSPTFDQLEADLRKRLRAVSFETIDVLRRS 479
14_NZE8    401 RLVNLLSQRLEGVRLRNHVASSGSSSSLESIGEDSVVGVSLSATAGSPLSSPTFDQLEADLRKRLRAVSFETIDVLRRS 479
15_RUS1    401 RLVNLLSQRLEGVRLRNHVASSGSSSSLESIGEDSVVGVSLSATAGSPLSSPTFDQLEADLRKRLRAVSFETIDVLRRS 479
16_SAF4    401 RLVNLLSQRLEGVRLRNHVASSGSSSSLESIGEDSVVGVSLSATAGSPLSSPTFDQLEADLRKRLRAVSFETIDVLRRS 479
17_SLV1    401 RLVNLLSQRLEGVRLRNHVASSGSSSSLESIGEDSVVGVSLSATAGSPLSSPTFDQLEADLRKRLRAVSFETIDVLRRS 479
18_USA12   401 RLVNLLSQRLEGVRLRNHVASSGSSSSLESIGEDSVVGVSLSATAGSPLSSPTFDQLEADLRKRLRAVSFETIDVLRRS 479
               *******************************************************************************
```

Figure S4. Alignment of pathway regulator DsAflR from 19 *D. septosporum* strains.

Amino acid changes compared to strain NZE10 are highlighted in blue (these sites also variant between AflR sequences of *D. septosporum, C. fulvum, Aspergillus parasiticus* and *A. nidulans;* (Chettri et al., 2013)) or in green (at sites conserved between those four species). The Zn_2Cys_6 zinc binuclear domain is highlighted in pink; the linker sequence thought to determine DNA-binding specificity in grey; the acidic glutamine-rich motif in yellow and C-terminal arginine residues implicated in AflJ binding in red.
