# Supplementary material for: Global population genomics of the forest pathogen Dothistroma septosporum reveal chromosome duplications in high dothistromin‐producing strains
Source: Mol Plant Pathol. 2019 Apr 1;20(6):784–99. doi: 10.1111/mpp.12791 (PMC6637865; doi:10.1111/mpp.12791)
Supplement: Supplementary file 5 — Fig. S5 Secondary structure predictions for AflR from D. septosporum NZE10 and ALP3. Pairwise alignment predicted by HHpred. The arrow indicates the location of the N349K polymorphism in ALP3. [file MPP-20-784-s005.pdf]

Fig. S5 Secondary structure predictions for AflR from *D. septosporum* NZE10 and ALP3. Pairwise alignment predicted by HHpred. The arrow indicates the location of the N349K polymorphism in ALP3.

```
Q ss_pred              CCccccccCCCCccCCcCCCCCCCChhhHhhcHHHHHhcCCCCCCCCCcHHHHHCCCccceecccCCCCC
Q 0_NZE10      1       MPESRGSESSGSTSRRASGTQHIATPKLKDSCTACATSKVKCSKDKPTCARCTRRGLTCDYGLSKRTGRT   70 (479)
Q Consensus    1       ~~~~~~~~~~~~~~~~~~~~~~~~~~KlR~SC~~CA~SKVrC~KeKPtCsRC~~rg~~C~Y~~srR~GR~   70 (479)
                       ||++||+.+...++............|.|..|..|...|++|.++||.|.||.+++..|+|...+|.|++
T Consensus    1       ~~~~~~~~~~~~~~~~~~~~~~~~~~r~~~ac~~c~~~k~~c~~~~p~c~~c~~~~~~~C~y~~~~r~~~~   70 (479)
T 1_ALP3       1       MPESRGSESSGSTSRRASGTQHIATPKLKDSCTACATSKVKCSKDKPTCARCTRRGLTCDYGLSKRTGRT   70 (479)
T ss_pred              CCcccCCCCCCCccccCCCCCCCCCcccchHHHHHHHhcCcCCCCCCCCcccchHHcCCcCccCcccCCCCC


Q ss_pred              CCcchhHHHHHHHHHHHHHHHHhhccccccccccccCCCCcccccCCcccccCCCCCcccCCcccccCCC
Q 0_NZE10      71      SHATAQKQQQQRQQQQQSQQEAADRRVSQSAVPVANMLPDVDEFISPMSLQESSILSPTLMPDLSTSGSQ   140 (479)
Q Consensus    71      ~~~~~~~~~~~~~~~~~~~~~~~~~~~~~~~~~~~~~~~~~~~~~~~~~~~~~~~~~~~~~~~~~~~~~~   140 (479)
                       +.+++.........+...+.+.++.+.+|+.+++.+..++..+|+.++++++++++++++++..++..++
T Consensus    71      ~~~~~~~~~~~~~~~~~~~~~~~~~~~~~~~~~~~~~~~~~~~~~~~~~~~~~~~~~~~~~~~~~~~~~~   140 (479)
T 1_ALP3       71      SHATAQKQQQQRQQQQQSQQEAADRRVSQSAVPVANMLPDVDEFISPMSLQESSILSPTLMPDLSTSGSQ   140 (479)
T ss_pred              cchHHHHHHHHHHHHHHHHHhhhhhhhhhcccccccCCCCCCccCCChhhccCCCCCcccCCccccccCC


Q ss_pred              CCcccccccHhhccCCCCCCCCCCCcchhhccccCCCCCchhHhhhccccccccCCCCCcccccccccccc
Q 0_NZE10      141 AGLDQWNSDLWSTMFAPNEPSSNNTPANTMQTHGGNGNDIDHLFNDLTSYSMLGEPTDTAMSNQHTHTFS   210 (479)
Q Consensus    141 ~~~d~~~~~~~~~~~~~~~~~~~~~~~~~~~~~~s~~~~~~~~~~~~~ls~~~~~~~~~~~~~~~s~~~~~~~~~   210 (479)
                       +++|+||.++|++.|+++++++++++++++.+.++.++++|++++|.++++|++++.+++.+++++++++|+
T Consensus    141 ~~~~~~~~~~~~~~~~~~~~~~~~~~~~~~~~~~~s~~~~~~~~~~~~s~~~~~~~~~~~~~~~~~~~~~~~   210 (479)
T 1_ALP3       141 AGLDQWNSDLWSTMFAPNEPSSNNTPANTMQTHGGNGNDIDHLFNDLTSYSMLGEPTDTAMSNQHTHTFS   210 (479)
T ss_pred              CChhHhccchHHhhcCCCCCCCCCCcchhHhcccCCCCCchhHhhhcCCchHhcCCCCCcccccccccccc


Q ss_pred              cCCCCCChhhhccchhccCCcccccccccccCCChhhhcCCCCCCcHHHHHHHHHHHHhcccccccCCCC
Q 0_NZE10      211 DTSSSISPFDQLTSVSDMGSQDFSNIDLSMTNCTPAIQQSVEPANCCLTVALGFMTQLCATASSSCTMPG   280 (479)
Q Consensus    211 ~~s~~~~~~~~~~~~~~~~~~~~~~~~~~~~~~~~~~s~p~~~~~~~~~~~~~~~~~~~~c~Cl~~Al~lL~~L~~~~~~~~sc~~~~   280 (479)
                       +++.+++.+++|+.++++++.+|++++.+.|+..+.........|+|+..||++|++|+.....+|..+.
T Consensus    211 ~~~~~~~~~~~~~~~~~~~~~~~~~~~~~~~~~~~~~s~~~~~~~~~~~~~~~~~~~~~~~~~~~~c~Cl~~al~~L~~L~~~~~~~~~c~~~~   280 (479)
T 1_ALP3       211 DTSSSISPFDQLTSVSDMGSQDFSNIDLSMANCTPAIQQSVEPANCCLTVALGFMTQLCATASSSCTMPG   280 (479)
T ss_pred              CCCCCCChHHhcccccccCcccccccccccCCCchhhccCCCCCcHHHHHHHHHHHHhccccccCCCCC


Q ss_pred              CCCCCCCCCcHHHHHHHHHHHHHHHHHHHhCCCCCCCHHHHHHHHHHHHHHHHHHHHHHHHHhcCCcchhcccc
Q 0_NZE10      281 SHNGNTTLPTIDSVITENRQIVDQIVKILECPCSHDEYLLTIVHLVVFKVMAWYAAAAREKPSLAEEINW   350 (479)
Q Consensus    281 ~~~~~~~~~~~sld~vL~~nk~ai~~~~~~iL~C~Cs~d~~ll~LlslI~~KIL~~Y~aaa~~~~~~~~~~~~~~~~   350 (479)
                       ........+.+|.||..||++|+.|..||+|.|..+.++++||++|++|||+||+++++......+...|
T Consensus    281 ~~~~~~~~~~~~~~~d~vL~~nk~ai~~~~~~iL~C~C~~d~~~l~Ll~~i~~kil~~Y~~~~~~~~~~~~~~~~~~~~~~~~~~~~~   350 (479)
T 1_ALP3       281 SHNGNTTLPTIDSVITENRQIVDQIVKILECPCSHDEYLLTIVHLVVFKVMAWYAAAAREKPSLAEEIKW   350 (479)
T ss_pred              CCCCCCCCccHHHHHHHHHHHHHHHHHHHCCCCcCCHHHHHHHHHHHHHHHHHHHHHHHHHhcCCcchhhccc


Q ss_pred              ccccCCCCCCCCCCccccCCCCeecceeCChhHHHHHHHHHHHHHHHHHHHHHHHHHHHHHHHHcccccchhcc
Q 0_NZE10      351 TDQQSGRPRSRTHSEEVLRFPPSIDGYSSDGSDNGRMAAQLVLSELHRVQRLVNLLSQRLEGVRLRNHVA   420 (479)
Q Consensus    351 ~~~~~sg~~~s~s~~~~~~~~~~~~~~~~iG~y~ld~~d~~~~~~~aqLVL~EL~rvqrLv~~L~~rl~~~~~~~~~~~~~~~~   420 (479)
                       .+..++...+.+........++.||+|.+|++++.+|++||||.|||||++||++|+++++..++++...
T Consensus    351 ~~~~s~~~~~s~~~~~~~~~~~~~~~~~~~~G~~~id~~~~~~~~~~~aqLVl~eL~rvq~lv~~L~~rl~~~~~~~~~~~~~~~~   420 (479)
T 1_ALP3       351 TDQQSGRPRSRTHSEEVLRFPPSIDGYSSDGSDNGRMAAQLVLSELHRVQRLVNLLSQRLEGVRLRNHVA   420 (479)
T ss_pred              ccccCCCCCCCCCCcccccCCCeecceeCCchHHHHHHHHHHHHHHHHHHHHHHHHHHHHHHHHcccccccccc
```

```
Q ss_pred             ccCCcccccccCCCcccccCcccCCCCCCCHHHHHHHHHHHHHHHHHHHHHHHHHHHhC
Q 0_NZE10      421 SSGSSSSLESIGEDSVVGVSLSATAGSPLSSPTFDQLEADLRKRLRAVSFETIDVLRRS  479 (479)
Q Consensus    421 ~~~~~~~~~~~~~~~~~~~~~~~~~~~p~s~~~l~qle~dLR~rLr~LS~~ii~~LR~~  479 (479)
                     ..+..+..++|++....+...+.+...|||..+|+|||.|||||||+||.|||++|||+
T Consensus    421 ~~~~~~~~~~~~~~~~~~~~~~~~~~~p~s~~~l~qle~dlr~rLr~ls~~ii~~Lr~~  479 (479)
T 1_ALP3       421 SSGSSSSLESIGEDSVVGVSLSATAGSPLSSPTFDQLEADLRKRLRAVSFETIDVLRRS  479 (479)
T ss_pred             cCCccccchhcCCCcccccCcccccCCCCCHHHHHHHHHHHHHHHHHHHHHHHHHHHhC
```
