# Supplementary material for: Global population genomics of the forest pathogen Dothistroma septosporum reveal chromosome duplications in high dothistromin‐producing strains
Source: Mol Plant Pathol. 2019 Apr 1;20(6):784–99. doi: 10.1111/mpp.12791 (PMC6637865; doi:10.1111/mpp.12791)
Supplement: Supplementary file 9 — Table S4 Single Nucleotide Polymorphisms (SNPs) in dothistromin genes, grouped by dothistromin gene loci. [file MPP-20-784-s009.pdf]

Table S4. Single Nucleotide Polymorphisms in dothistromin genes, grouped by dothistromin gene loci

New gene name	Dotse1 Protein ID[a]	Amino acids	Unique missense SNPs[b]	Unique synonymous SNPs[b]	Selection dN/dS mean[c]	% ID[d] Ap	% ID An	Predicted or known function
Ver1	75411	264	1	28	0.0199	79.1	79.2	NAD(P) reductase
DotB	75412	415	16	38	0.2035	-	24.0	peroxidase
DotC	75413	581	13	59	0.0779	31.2	-	MFS transporter
PksA	48345	2400	37	113	0.1653	55.1	58.2	polyketide synthase
CypX	139960	512	6	28	0.0886	58.9	61.6	P450 monooxygenase
AvfA	75546	285	9	18	0.1651	49.3	44.9	NAD(P) reductase
EpoA	57187	421	15	33	0.1513	-	-	Epoxide hydrolase
MoxY	75547	627	9	39	0.1034	55.4	50.6	flavin-binding monooxygenase
AflR	75566	480	6	22	0.1087	27.5	30.4	Regulatory protein
AflJ	57214	457	18	29	0.2424	37.1	40.8	Methyltransferase
Est1	75609	329	9	37	0.0899	29.6	27.8	Esterase (alpha/beta hydrolase)
OrdB	75648	268	5	14	0.1012	54.1	45.3	NAD(P) reductase
AvnA	57312	526	16	79	0.0845	57.5	58.3	P450 monooxygenase
HexB	181128	1905	35	131	0.1380	52.2	46.0	Fatty acid synthase
HexA	75653	1693	34	93	0.1845	55.4	45.5	Fatty acid synthase
HypC	66978	186	12	15	0.2864	35.2	47.9	Anthrone oxidase
VbsA	75656	648	5	48	0.0548	72.3	73.1	VerB synthase (cyclase)
Nor1	75691	269	7	28	0.0662	59.7	58.5	NAD(P) reductase
AdhA	48495	307	5	41	0.0239	58.1	60.6	Alcohol dehydrogenase
VerB	75692	521	5	57	0.1765	67.1	67.6	Desaturase (P450 monooxygenase)

Row shading indicates groupings of genes (top to bottom) in loci 1-6 (Chettri et al 2013, Fungal Genetics and Biology, 51, 12-20).

[a]Protein identification (accession; PID) numbers refer to those at (http://genome.jgi.doe.gov/Dotse1/Dotse1.home.html). The PID numbers shown are the searchable JGI gene models; updated Dotse1 PID numbers for improved gene models are *HexA* 66976, *PksA* 192192, *Ver1* 192193, *HypC* 75655.

[b]Number of unique SNPs (exon sequence only) compared to NZE10.

[c]Mean of all pairwise comparisons between each genome and the NZE genome, calculated using CodeML.

[d]Percentage amino acid identities (% ID) to *A. parasiticus* (Ap) and *A. nidulans* (An) AF/ST genes determined by CLUSTALW whole sequence alignment.
