# Supplementary material for: Global population genomics of the forest pathogen Dothistroma septosporum reveal chromosome duplications in high dothistromin‐producing strains
Source: Mol Plant Pathol. 2019 Apr 1;20(6):784–99. doi: 10.1111/mpp.12791 (PMC6637865; doi:10.1111/mpp.12791)
Supplement: Supplementary file 10 — Table S5 Deleted genes on Dothistroma septosporum chromosome 12. [file MPP-20-784-s010.pdf]

Table S5. Deleted genes on *Dothistroma septosporum* chromosome 12

JGI protein ID	Times Deleted[a]	Start[b]	End	FM[c]	Early	Middle	Late	Function prediction[d]
57306	12	1013331	1014396	72.82	21.46	30.29	75.92	protein kinase
29200	12	1014846	1015758	1.61	0.00	4.04	17.45	-
75644	12	1015931	1017521	23.11	4.32	16.52	22.93	-
181103	12	1017726	1018777	31.96	16.25	19.12	29.70	NAD+ binding
57307	13	1019402	1020382	2.12	0.00	0.00	31.93	-
39202	14	1020670	1025486	3.65	3.28	2.25	24.54	protein kinase
39203	14	1025626	1026415	0.44	0.00	2.04	23.66	-
39204	14	1028725	1029975	1.29	0.00	1.10	3.04	nucleic acid binding
75646	14	1030345	1033907	32.28	44.43	35.74	31.84	GTP metabolism
139618	14	1103995	1104180	0	0	0	0	-

[a]Number of genomes (out of 18) in which the gene was deleted. These inferences from CNVnator data were confirmed by visualisation of alignments, except visualisation suggestded one more deletion of 29200, 75644 and two more deletions of 181103.

[b]Position of the gene (start and end) on *D. septosporum* NZE10 chromosome 12 (www.genome.jgi.doe.gov/Dotse1)

[c]Gene expression (reads per kb per million) in *D. septosporum* NZE10 in culture (FM) or *in planta* (early, mid, late stages of disease) from Bradshaw et al (2016) Molecular Plant Pathology 17, 210-224..

[d]Based on GO terms, where available.
