# Supplementary material for: Global population genomics of the forest pathogen Dothistroma septosporum reveal chromosome duplications in high dothistromin‐producing strains
Source: Mol Plant Pathol. 2019 Apr 1;20(6):784–99. doi: 10.1111/mpp.12791 (PMC6637865; doi:10.1111/mpp.12791)
Supplement: Supplementary file 12 — Table S7 (a) Polymerase Chain Reaction (PCR) primers used for verification of 5:13 translocation (b) Primers used for copy number variant (CNV) verification (quantitative PCR [qPCR]). [file MPP-20-784-s012.pdf]

Table S7a. PCR primers used for verification of 5:13 translocation

Primer pair[a]	Start[b]	End[b]	Length bp[c]	Primer sequence 5’ to 3’	In-house number
1_3	1459101	1459967	866	1_AGTCCTGCTGTTTCCGCTAC<br>3_CCCAGTGTCAGAGAGGAGGA	1715<br>1717
5_7	717616	718513	897	5_CATCAAGCAAGAGCAGCTGC<br>7_GAGATGGCGTGAGTCGAGAG	1719<br>1721
1_5	1459101	718513	1289	1_AGTCCTGCTGTTTCCGCTAC<br>5 CATCAAGCAAGAGCAGCTGC	1715<br>1719
3_7	1459967	718513	757	3_CCCAGTGTCAGAGAGGAGGA<br>7_GAGATGGCGTGAGTCGAGAG	1717<br>1721

[a]See Figure 2 for schematic of primer positions
[b]Start and end indicate positions of the primers (amplicon ends) on chromosome 12 of *D. septosporum* NZE10 (http://genome.jgi.doe.gov/Dotse1/Dotse1.home.html)
[c]Length of the PCR amplicon

Table S7b: Primers used for copy number variant verification (qPCR)

Primer	Target gene	In-house lab No	5' to 3' sequence
MR-18_16S F	16S rRNA	1963	AAATCATCATGCCCCTTATG
MR-19_16S R		1964	CGATTACTAGCAATTCCGACT
Ds72297 F	Ds72297	1965	GCACACTCACCAACAAGATG
Ds72297 R		1966	CACGAGCCCATAGAGATAACC
Ds72201 F	Ds72201	1967	TCAAATCACCACCAGAATAGC
Ds72201 R		1968	TAG ATGAGCCGATGACTACTT
Ds72172 F	Ds72172	1969	CATTCGGCATCGCAGGTAAG
Ds72172 R		1970	CTCACGCCATCTATTGGTTCTTG
Ds72010 F	Ds72010	1971	GATGCCGTCCTCCAATCTATGA
Ds72010 R		1972	ATAGTCAGTGCCTCGCCAAA
Ds75320 F	Ds75320	1973	ATCGTTGGTGATGACTTGACT
Ds75320 R		1974	GGATAGCCTCGGAGATGGTA
Ds75922 F	Ds75922	1975	CCGACACATACTGATACTAC
Ds75922 R		1976	GAAGCAGATAAGGATGACAT
Ds161036 F	Ds161036	1977	CTTCAAGGCTGTTCCCATCC
Ds161036 R		1978	CCACTCATCGGTTCTCTTATTCG
Ds75737 F	Ds75737	1979	TACAAGACTACCTTCGCCATC
Ds75737 R		1980	ACA GAGCAATGTCTCGACTTA
Ds75967 F	Ds75967	1981	ATATCATCTGGACGGAATCAA
Ds75967 R		1982	CTCCCCTTCATACACTCATT
Ds71743 F	Ds71743	1983	AAGTCTTGGTGGATATACAT
Ds71743 R		1984	CAGGACATTGATATTGTTCA
Ds29553 F	Ds29553	1985	TAAGCGAGGAGATTGAGGAT
Ds29553 R		1986	TGAGCGAGGATGTAGAAGAT
Ds75914 F	Ds75914	1987	GAAGGCGTGTTGTCTGTTGA
Ds75914 R		1988	GTACCATGCCACCATCTTGAAT
Ds71757 F	Ds71757	1989	TGAGCATTAACAGCAACACT
Ds71757 R		1990	CCGATGAGGATGTCTTGAAG
Ds158381 F	*DsEcp2-1*	1991	TGGCTGACTCGTCGTATAACC
Ds158381 R		1992	AGATACCGCCCAGATGTTGTT
DsDotC F	*DsDotC*	1993	CACTCCTTCGTCTTCATCTC
DsDotC R		1994	CTGACACCTGTGGACAAC
DsDotB F	*DsDotB*	1995	CAATACTCCGTGGCGAACAA
DsDotB R		1996	AGTGGTTGGCGAAGAGGTA
